# A spheroid whole mount drug testing pipeline with machine-learning based image analysis identifies cell-type specific differences in drug efficacy on a single-cell level

**DOI:** 10.1186/s12885-024-13329-9

**Published:** 2024-12-18

**Authors:** Mario Vitacolonna, Roman Bruch, Richard Schneider, Julia Jabs, Mathias Hafner, Markus Reischl, Rüdiger Rudolf

**Affiliations:** 1 CeMOS, Mannheim University of Applied Sciences, 68163 Mannheim, Germany; 2 Institute of Molecular and Cell Biology, Mannheim University of Applied Sciences, 68163 Mannheim, Germany; 3 Institute for Automation and Applied Informatics, Karlsruhe Institute of Technology, 76344 Eggen-stein-Leopoldshafen, Germany; 4 Merck Healthcare KGaA, 64293 Darmstadt, Germany; 5 Institute of Medical Technology, Medical Faculty Mannheim of Heidelberg University, Mannheim University of Applied Sciences, 68167 Mannheim, Germany

**Keywords:** 3D co-culture, 3D drug testing, Deep-learning image analysis, Tumor microenvironment, Single-cell analysis, Drug resistance

## Abstract

**Background:**

The growth and drug response of tumors are influenced by their stromal composition, both in vivo and 3D-cell culture models. Cell-type inherent features as well as mutual relationships between the different cell types in a tumor might affect drug susceptibility of the tumor as a whole and/or of its cell populations. However, a lack of single-cell procedures with sufficient detail has hampered the automated observation of cell-type-specific effects in three-dimensional stroma-tumor cell co-cultures.

**Methods:**

Here, we developed a high-content pipeline ranging from the setup of novel tumor-fibroblast spheroid co-cultures over optical tissue clearing, whole mount staining, and 3D confocal microscopy to optimized 3D-image segmentation and a 3D-deep-learning model to automate the analysis of a range of cell-type-specific processes, such as cell proliferation, apoptosis, necrosis, drug susceptibility, nuclear morphology, and cell density.

**Results:**

This demonstrated that co-cultures of KP-4 tumor cells with CCD-1137Sk fibroblasts exhibited a growth advantage compared to tumor cell mono-cultures, resulting in higher cell counts following cytostatic treatments with paclitaxel and doxorubicin. However, cell-type-specific single-cell analysis revealed that this apparent benefit of co-cultures was due to a higher resilience of fibroblasts against the drugs and did not indicate a higher drug resistance of the KP-4 cancer cells during co-culture. Conversely, cancer cells were partially even more susceptible in the presence of fibroblasts than in mono-cultures.

**Conclusion:**

In summary, this underlines that a novel cell-type-specific single-cell analysis method can reveal critical insights regarding the mechanism of action of drug substances in three-dimensional cell culture models.

**Supplementary Information:**

The online version contains supplementary material available at 10.1186/s12885-024-13329-9.

## Background

The dynamic interplay between tumor cells and their microenvironment significantly influences tumor progression and response to therapeutic interventions. Among the various stromal cell types, fibroblasts have emerged as key players in modulating tumor growth and drug resistance through a variety of mechanisms. They modulate the extracellular matrix, impacting drug delivery and penetration, and can modify tumor cells’ drug responses via paracrine signaling. This dynamic interplay between fibroblasts and tumor cells can lead to drug resistance, presenting a significant challenge in effective cancer therapy [1–5]. To dissect these complex interactions, advanced 3D in vitro models are helpful, since they enable the study of multidimensional interactions and cellular behavior that closely resemble those of actual tumor tissue [6–9] and serve as robust platforms for evaluating drug efficacy in the presence or absence of stromal cells [10, 11]. While several 3D co-culture models of tumor cells and fibroblasts have been developed, many of them come with limitations. For example, although microarray chip-based models have been used to mimic metabolic interplay and allow for mechanistic analyses, they often lack the structural complexity and long-term stability needed to fully replicate tumor-stroma interactions [12]. Similarly, microfluidic chip models offer innovative ways to study cancer progression and cell-cell interactions, but their ability to fully recreate the tumor microenvironment and consistently scale up for drug testing remains limited [13–18]. Other approaches, such as self-assembled peptide scaffolds [19] and microencapsulated 3D co-cultures [20], while providing more realistic tumor modeling face challenges related to reproducibility, scalability, and the accurate representation of dynamic cell behaviors over time. For instance, hanging drop techniques used with non-small cell lung cancer and fibroblast co-cultures are difficult to integrate with high-throughput drug screening applications [21]. The model introduced in this study addresses several of these limitations. Our approach incorporates a deep-learning based analysis combined with the ability to distinguish between tumor cells and fibroblasts through both, nuclear morphology and collagen-1 secretion. This allows for a more accurate reflection of the cellular heterogeneity and dynamic interactions present in the tumor microenvironment. Additionally, our model improves upon existing techniques by offering better scalability and compatibility with high-throughput analysis, making it a more versatile tool for studying drug efficacy and tumor progression in 3D co-cultures.

However, tumors are typically heterogeneous structures, comprising various cell types that exhibit distinct responses to cytostatic drugs. This heterogeneity underscores the importance of evaluating the effects of these drugs on each specific cell type during drug testing. Tumor cells, fibroblasts, immune cells, and other constituents of the tumor microenvironment may each respond differently to a particular drug, thereby affecting the overall treatment efficacy [22–26]. Therefore, understanding the dynamics of these differential responses at the cellular level is essential for developing more effective therapies, where treatments are based on the specific characteristics of a patient’s tumor. To this end, detailed single-cell analyses are necessary for comprehensive drug testing to gain a deeper understanding of the complex interactions within the tumor microenvironment and for evaluating cell-type-specific responses to the therapeutic substances. Although recent studies using 3D-spheroid co-cultures have revealed changes in growth kinetics and drug responses in tumor models, they mainly concentrated on the general effects within 3D cultures. This approach often neglects the nuanced, cell-type-specific interactions and responses observable at the single-cell level, highlighting the need for a comprehensive analysis of drug treatments on entire 3D tumor samples on single-cell level to overcome the limitations of traditional slide-based methods.

**Fig. 1:**
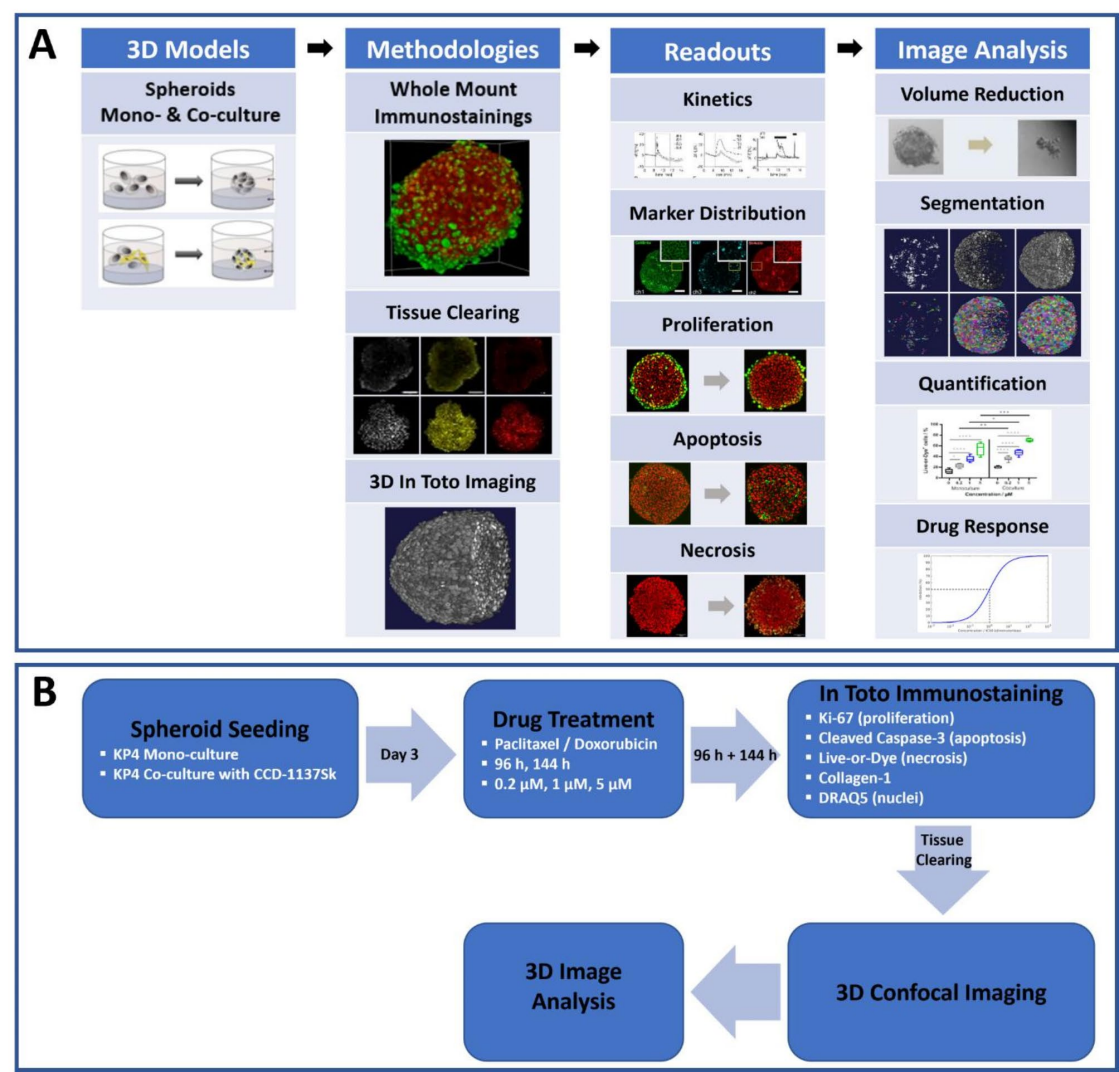
A pipeline for quantitative analysis of cell-type specific drug effects in 3D-cell culture models. (**A**) Workflow of the 3D drug testing pipeline. The pipeline has a modular structure and includes various segments, such as the setup of 3D-models in mono- and co-culture, as well as the implementation of whole mount 3D-stainings. To increase the penetration depth of whole mount microscopy and to allow the visualization of the entire sample, a tissue clearing step followed by 3D confocal microscopy was integrated into the pipeline. Depending on the research question, a suitable marker panel can be selected to stain the target molecules in toto, which can subsequently be quantified using a combination of classical and AI-based image analysis methods. (**B**) Experimental design overview. The effect of drug treatment on KP4 tumor spheroids in mono- and co-culture with fibroblasts was investigated. The study included the preparation of KP4 tumor spheroids in mono- and co-culture with CCD-1137Sk fibroblasts, treatment with two chemotherapeutic drugs (paclitaxel and doxorubicin) for 96 and 144 h at three different concentrations, whole mount immunostaining with different markers to identify proliferating, apoptotic and necrotic cells as well as collagen-1 secretion. This was followed by in toto 3D-imaging using confocal microscopy and quantification by 3D-image analysis

The present study aims to bridge this gap by providing an in-depth analysis of drug treatment effects on whole mount 3D spheroid samples to provide a more detailed understanding of the interplay between tumor cells and fibroblasts within the tumor microenvironment. To achieve this, we developed a 3D drug-testing pipeline using KP4 tumor spheroids in both mono- and co-culture with fibroblasts. The pipeline features spheroid whole mount staining, optical tissue clearing, and 3D confocal microscopy. Mono- and co-culture spheroids were exposed to four different doses of paclitaxel or doxorubicin for periods of 96 and 144 h and analyzed with a marker panel to identify proliferative, apoptotic, and necrotic cells across full samples. Additionally, we utilized custom-trained convolutional neural network (CNN)-based 3D-image analysis tools for detailed evaluation of the cell-type-specific impact of paclitaxel and doxorubicin at the single-cell level (see Fig. 1A for a quick overview of the drug testing pipeline and Fig. 1B for experiment design).

## Methods

### Cell culture and spheroid generation

To prepare spheroids in mono- and co-culture, the appropriate number of cells were seeded on Ultra Low Attachment (ULA) 96-well U-bottom plates (Corning) in the appropriate medium and centrifuged at 20 g for 2 min. The decision on seeding density and ratio was based on preliminary experiments to generate growth curves, which allowed us to determine the appropriate seeding densities. These densities were selected to ensure that the spheroids would reach the desired diameters after three days of cell culture. All cells were thawed and cultured in a humidified incubator at 37 °C with 5% CO_2_ fumigation for at least 3 passages before starting the assays. Spheroids were cultured for 3 days before drug treatment. KP4 pancreatic ductal cell carcinoma cells (Riken Cell Bank, passage number between 16 and 21) were cultured in DMEM/F12 (Gibco) supplemented with 10% FBS (fetal bovine serum) and 1% pen/strep (Capricorn). For KP4 spheroid mono-culture generation, cells were detached using trypsin/EDTA (Capricorn), seeded onto 96-well ULA plates at a concentration of 5 × 10^2^ cells per well and cultured for three days. CCD-1137Sk human foreskin fibroblast cells (ATCC, passage numbers between 11 and 16) were cultured in Iscove’s modified Dulbecco’s medium (IMDM, Capricorn) supplemented with 10% FBS and 1% pen/strep. For CCD-1137Sk spheroid mono-culture generation, cells were detached using trypsin/EDTA and seeded onto 96-well ULA plates at a concentration of 1.5 × 10^3^ cells per well and cultured for 3 days. For cocultures, KP4 tumor cells were mixed 1:3 with CCD-1137Sk fibroblasts. Briefly, 5 × 10^2^ tumor cells were mixed with 1.5 × 10^3^ CCD-1137Sk fibroblast cells and cultured for three days in 96-well ULA plates with an equal volume of the corresponding medium. We assessed the reproducibility of our 3D spheroid cultures by calculating the coefficient of variation (CV) between replicates. The CV for spheroid diameter after 3 days of culture was on average 4% for the KP4 mono-cultures, 6% for the CCD-1137Sk fibroblasts, and 8% for the co-cultures, indicating a high level of consistency and robustness in our cell cultures.

### Drug treatments

Paclitaxel and doxorubicin were prepared as stock solutions of 10–100 mM in dimethyl sulfoxide of tissue culture grade (DMSO; Sigma-Aldrich), with a final DMSO concentration of 0.1% in the media. The concentrations of paclitaxel and doxorubicin (0.2, 1, and 5 µM) were selected based on literature reviews of clinically relevant doses for 3D in vitro models, ensuring both sublethal and lethal ranges to capture diverse cellular responses. These concentrations were extensively tested in previous drug screenings, confirming their suitability for clinical relevance and robustness in our 3D model. After 3 days in culture, spheroids in mono- and co-culture were treated either with paclitaxel (Sigma-Aldrich) or doxorubicin (Selleckchem) at 3 different concentrations (0.2, 1 and 5 µM) for 96 and 144 h in 96-well ULA plates as previously described [27]. Briefly, a 2x desired concentration of the drug was first prepared in media. This was followed by a 1:1 dilution in the appropriate media to give the desired final concentration. Controls received the same amount of vehicle (DMSO) as the drug-treated cells (0.1% v/v).

### Whole mount immunostaining and optical clearing

Whole mount immunostainings were performed as previously described [28, 29]. Briefly, spheroids were transferred to Eppendorf tubes, washed once with phosphate buffered saline (PBS, Sigma Aldrich) and fixed with 4% paraformaldehyde (PFA, Carl Roth) for 1 h at 37 °C, followed by two washes with PBS containing 1% FBS for 5 min each. To remove traces of fixative, spheroids were quenched with 0.5 M glycine (Carl Roth) in PBS for 1 h at 37 °C with gentle shaking. Spheroids were then incubated for 30 min in penetration buffer containing 0.2% Triton X-100, 0.3 M glycine and 20% DMSO (all Carl Roth) in PBS to enhance the penetration of antibodies and nuclear stains. Spheroids were then incubated in blocking buffer (0.2% Triton X-100, 1% BSA, 10% DMSO in PBS) for 2 h at 37 °C with gentle shaking. After blocking, samples were incubated with primary antibodies overnight (ON) at 37 °C with gentle shaking. Primary antibodies were diluted in antibody buffer (0.2% Tween 20, 10 µg/ml heparin (both Sigma-Aldrich), 1% BSA, 5% DMSO in PBS) at the following concentrations: anti-Ki-67 (Merck, rabbit polyclonal antibody) 1:300, anti-Cleaved Caspase-3 (CellSignaling, rabbit polyclonal antibody) 1:400, and anti-collagen-1 (Rockland, rabbit polyclonal antibody) 1:200.

Samples were then washed 5 x for 10 min each in wash buffer (0.2% Tween-20, 10 µg/mL heparin, 1% BSA) and stained with secondary antibodies and nuclear dyes ON at 37 °C with gentle shaking. The appropriate secondary antibodies and nuclear dye were diluted in antibody buffer at the following concentrations: donkey anti-rabbit IgG (H + L) Alexa Fluor^®^488 1:800 and DRAQ5™ 1:1000 (all Invitrogen). Samples were then washed 5 x for 10 min in washing buffer with gentle shaking and then cleared with 88% glycerol. Glycerol-based RI adjustment was performed according to Nürnberg et al. [30] by immersion of stained spheroids in an aqueous solution of 88% glycerol (RI 1.459) ON at RT with gentle shaking, followed by mounting on 18 well µ-slides (Ibidi) in the same solution. After mounting, spheroids were kept in the microscope room for several hours to allow for temperature adjustment.

Dead cells were stained with the fixable necrotic marker Live-or-Dye NucFix™ Red Staining Kit (Biotium) according to the manufacturer’s instructions. Briefly, spheroids were transferred to Eppendorf tubes, washed with 1 x Hanks balanced salt solution (HBSS) and incubated in Live-or-Dye solution (1:1000) for 2 h at 4 °C in the dark with gentle shaking. The samples were then washed 3 x for 10 min in 1x HBSS and fixed with 4% paraformaldehyde for 1 h at 37 °C, followed by two washes with PBS containing 1% FBS for 5 min each and quenched with 0.5 M glycine in PBS for 1 h at 37 °C with gentle shaking. The samples were then washed 3 times for 10 min and cleared with 88% glycerol.

### Immunofluorescence staining on cryosections

Ten KP4 spheroids with a diameter of 500 μm were collected in an Eppendorf tube for cryosectioning. After being washed twice with PBS, they were fixed with 4% paraformaldehyde in PBS for 30 min at room temperature. Following this, the spheroids were incubated in 15% sucrose (Carl Roth) in PBS overnight at 4 °C, then in 25% sucrose in PBS again overnight at 4 °C. Finally, they were embedded in Tissue-Tek Cryomolds using OCT (Leica Biosystems). 5-µm thick sections were prepared using a CM-1950 cryostat (Leica Biosystems). Cryosections with diameters of 50, 250, and 500 μm were chosen. All cryosections were permeabilized with 0.1% Triton X-100 (Carl Roth) in PBS, then blocked with 2% BSA in PBS before being stained with either anti-Ki-67 (1:300) or anti-Cleaved Caspase-3 (1:400) for 1 h at room temperature. Samples were washed 3 x with PBS containing 1% FBS, followed by secondary antibody and nuclei staining using donkey anti-rabbit IgG (H + L) Alexa Fluor^®^488 (1:800) and DRAQ5 (1:1000). Finally, sections were washed 3 x with PBS/1% FBS, mounted with Mowiol (Carl Roth) and imaged using a confocal microscope (SP8, Leica).

### Comparison of immunostainings on whole mount and cryosections using CellProfiler

To assess the equivalence between whole mount and classical immunofluorescence staining protocols on sectioned samples, we performed a comparison of 3D in toto with immunostainings on cryosections. Cryosections of 5 μm thickness were prepared from KP4 spheroids and sections of 50, 250, and 500 μm diameter, as well as whole mount KP4 spheroids with a diameter of 500 μm were immunostained in parallel with anti-Ki-67, anti-Cleaved Caspase-3 and DRAQ5 (*n* = 6 for each method) as described above. Whole mount samples were cleared with 88% glycerol and imaged in 3D using confocal microscopy to obtain optical tissue sections. To make physical and optical sections comparable, 5 focal planes with a z-step size of 1 μm for each corresponding diameter of the cryosections were selected, combined as maximum z-projections and the absolute number of nuclei, Ki-67^+^ and Cleaved Caspase-3^+^ cells were quantified using CellProfiler [31]. Briefly, image stacks of physical sections and corresponding optical confocal sections were exported to ImageJ software. Multichannel stacks were split to continue with single-channel stacks of each marker separately and processed to maximum intensity projections using Fiji. Background was subtracted from all images by adjusting the lower and upper display range units using the Brightness/Contrast option. Images were transferred to CellProfiler for automated cell counting. The images were smoothed with a 5-pixel wide Gaussian filter and segmented using the *IdentifyPrimaryObjects* module using the Otsu method with two-class thresholding and an object diameter between 5 and 30 pixels to exclude cell debris. The number of nuclei, proliferating and apoptotic cells were quantified and exported in both tiff format and an Excel spreadsheet.

### 3D image acquisition using confocal microscopy

All 3D cultures were imaged using an inverted Leica TCS SP8 confocal microscope (Leica Microsystems CMS, Mannheim, Germany) equipped with an HC PL APO 20× /0.75 IMM CORR objective, 405 nm, 488 nm, 561 nm, and 633 nm lasers and Leica Application Suite X software. All image stacks were acquired with comparable settings, using Immersion Type F (Leica Microsystems, RI 1.52) as immersion fluid, with a resolution of 1024 × 1024 pixels, a z-step size of 1 μm, a laser intensity of 1–2% and a gain setting of 600 V to avoid overexposure of pixels. All image stacks were acquired with z-compensation to compensate for depth dependent signal loss.

### 3D nuclei segmentation comparison utilizing two conventional methods and a convolutional neuronal network (CNN)

We compared the nuclear segmentation efficacy of the pre-trained, deep-learning-based segmentation network with two conventional open-access software packages based on 3D watershed, which were previously published. We used 3D confocal stacks from untreated co-cultured KP4 spheroids with a diameter of 300 μm and processed them initially using Fiji. Pre-processing included the cropping of images, correcting the background, and exporting as tiff files. Conventional method 1 included an image analysis pipeline based on Mathematica (Mathematica 11.1, Wolfram Research Inc.) [32]. The median filter range was set to 3 pixels, the local threshold range was set to 10 pixels, and the hole filling range was set to 1 pixel. For seed detection, Laplace of Gaussian (LoG) was chosen with a seed range between 9 and 25 pixels. The other parameters were used by default. The results of the initial and final segmentation and the detected seed positions for different planes in xy and zy were displayed in real-time to increase the accuracy of the segmentation results. The post-segmentation data were exported as different 3D stacks in tiff format and as an XLSX file with quantitative results. For conventional method 2, we utilized OpenSegSPIM, an automated quantitative analysis tool for 3D microscopy data [33]. The parameter settings for the median filter and noise removal size were set automatically after the average nucleus size was measured using the built-in module. For the detection step, we selected shape-based detection with a sensitivity of 1 and started the segmentation process using shape-based segmentation. The segmentation masks were exported as different 3D stacks in tiff format and as an Excel sheet with quantitative results. The CNN segmentation was performed by a deep-learning model presented by Scherr et al. [34]. The structure of the model and the data processing routines were adapted to allow for a direct 3D processing of the data. For training and inference, the images were sliced into patches of size 32 × 128 × 128 px^3^ (z, y, x). Training was performed with the ranger optimizer, an initial learning rate of 1 × 10^− 3^ and a batch size of three. Other training parameters were consistent with the ones used in [34]. The development of a substantial deep-learning training dataset for 3D data presents significant challenges. The extra dimension, relative to 2D data, substantially lengthens labeling time. Additionally, distinguishing nuclei in high-density areas is complex and labor-intensive, owing to reliance on 2D visualizations. Therefore, four synthetic 3D spheroid images, generated as described in [35] were used for model training. Quantitative analysis of the segmentation performance was conducted with the segmentation and detection measures used in the cell tracking challenge [36]. As ground truth, an image patch of size 32 × 128 × 128 px^3^ was manually annotated by a biological expert. The image patch was extracted from the 5 images.

### Downstream image analysis

Quantitative analysis was performed using dedicated Python scripts. All steps described here are part of the spheroid analysis Python script and are processed in an automatic manner. The script was designed to extract both, intensity and morphological features, of individual nuclei. Intensity features were calculated for images in their raw form as well as after application of a Gaussian filter. To mitigate the impact of noise and background signals, a user-defined intensity threshold was established to discern foreground signals. Subsequently, the image was processed with a Gaussian kernel, considering only voxel intensities above this threshold for the calculation of mean signal intensity. For each nucleus, statistical metrics such as mean, median, maximum intensity, the 95th percentile, and standard deviation of intensity were determined. These metrics were also derived for the region surrounding each nucleus. The key steps, the most important metrics generated with the Python-based 3D analysis script and additional information can be found online on GitHub.

Prior to further analysis, the spheroid images and deep-learning segmentation results were scaled in the z-direction to achieve isotropic resolution. Segmented nuclei with a volume smaller than 300 µm^3^ and larger than 3000 µm^3^ were considered as debris or segmentation errors and were excluded from further analysis. The nuclei volumes were then extracted from the segmentation masks using the `regionprops` function from the scikit-image Python package. Both Ki-67 and the necrosis marker Live-or-Dye were located within the cell nuclei, enabling their clear association with specific cells. Two support vector classification models were used to determine whether a cell was positive for Ki-67 or Live-or-Dye, respectively. The features used for the Ki-67 classification model were the average intensities in the nuclei and Ki-67 channel within the nucleus and the mean intensity in the Ki-67 channel within the proximate outer region of the nucleus. The outer region was defined by performing four binary dilations of the nuclei segmentation mask, using a structuring element with a connectivity of 1. The initial nucleus mask and all other nuclei masks were excluded from the proximate outer region. The features used for the Live-or-Dye classification model were the mean intensities in the nuclei and the Live-or-Dye channel within the nucleus. The Ki-67 classification model was trained on 201 manually annotated cells. The performance of this model was tested using 329 manually classified cells from two additional image stacks, with an accuracy of 97.26%. The Live-or-Dye classification model was trained on 85 manually classified cells. The performance of the model was evaluated using 73 labeled cells from an additional image and achieved an accuracy of 94.52%. To calculate the relative number of proliferative and necrotic cells, values of the Ki-67^+^ and Live-Or-Dye^+^ cells were divided by the total number of nuclei counted.

In the later stages of apoptosis, the precise attribution of caspase-3 activity to individual cells poses a significant challenge due to the dense clustering of these cells. To address this issue for quantification, the approach adopted involved aggregating the pixel intensity within the cleaved caspase-3 channel across the entire set of images. Subsequently, this aggregated signal was normalized by dividing it by the total count of nuclei present in the images. This methodology ensures a more accurate representation of cleaved caspase-3 activity on a per-cell basis, despite the spatial constraints imposed by cell clustering.

The quantification of spheroid characteristics such as volume, density, and spatial distribution of nuclei within the spheroid required a comprehensive segmentation of the entire spheroid. This process was initiated with the segmentation of nuclei, which served as a foundational step for subsequent measurements. Upon achieving segmentation of the spheroid, we proceeded to calculate its volume and the average density of nuclei within. To close the holes between nuclei without enlarging the spheroid’s segmented volume, a two-step process was employed: 40 iterations of binary dilation, followed by 40 iterations of binary erosion, using a structuring element characterized by a connectivity of 1. This procedure ensured the closure of inter-nuclear spaces without increasing the spheroid segmentation size. Subsequently, any remaining voids within the spheroid segmentations were filled to achieve a continuous structure. On occasion, certain cellular structures appeared as detached fragments, forming isolated entities. In these cases, only the largest contiguous structures were considered for analysis. We further analyzed the inter-nuclei spaces, herein referred to as voids, which represent the volume between individual nuclei of the whole spheroid. The proportion of these voids relative to the overall volume of the spheroid was meticulously calculated, offering insights into the spatial arrangement and density of the cellular components within the spheroid. The density of the nuclei was then calculated by correlating the total number of nuclei to the spheroid’s volume, providing an index of cellular compactness. The determination of the largest equivalent diameter within the xy-planes of the spheroid was based on the identification of the diameter of a circle that would encompass an area equivalent to that of the segmented xy-plane, thereby offering a geometric perspective on the spheroid’s spatial dimensions. Moreover, the script calculated the distance from the centroid of each nucleus to both the spheroid’s center and the nearest point on the spheroid’s hull. It also identified the specific hull on which each nucleus was located.

For 3D-shell analysis, the segmented spheroids were partitioned into 3 concentric equi-volumetric shells (inner, middle, and outer shell), each comprising an equal third of the total volume. These regions were delineated by sequentially applying binary erosion operations until the remaining volume was reduced to two-thirds and one-third of its original volume, respectively. For each shell, the nuclei count, nuclei density, and the average volume of nuclei were calculated automatically. Optionally, one can use the software´s GUI to additionally choose segmentation masks (e.g., Ki-67 segmentation masks can be selected to calculate the spatial distribution of Ki-67^+^ cells within the three shells) for the 3D-shell analysis.

### Discrimination of the fibroblast subpopulation in co-cultures using deep-learning based classification

Due to the lack of a highly specific fibroblast marker, a direct distinction between fibroblasts and tumor cells in co-cultures using immunofluorescence markers was not feasible. Therefore, we developed an approach that allowed us to clearly differentiate fibroblasts from tumor cells based on two key characteristics. First, fibroblast nuclei differred significantly in size and morphology compared to tumor cells. On average, fibroblast nuclei were smaller and elongated, whereas KP4 tumor cells showed considerably larger and rounder nuclei. However, since tumor cells in late mitotic phases can also exhibit smaller, more elongated nuclei, which can be easily misinterpreted as fibroblast nuclei, a discrimination based just on the difference in size was not sufficient and led to biased results. Nevertheless, it is well-known that fibroblasts secrete collagen-1, whereas KP4 mono-cultures do not [37, 38]. Therefore, we developed a deep-learning classification model that utilizes nuclear size, morphology, and collagen-1 staining to accurately distinguish between the two cell types. For methodological validation, fibroblasts stained with CellTracker were tracked within co-cultured spheroids. This process involved incubating 1.5 × 10^3^ fibroblasts with CellTracker Green (Life Technologies) for 45 min, following the manufacturer’s instructions. The CellTracker-labeled fibroblasts were subsequently seeded either in mono- or in co-culture with 5 × 10^2^ KP4 tumor cells in ULA plates and cultured for 3 days to form spheroids. The spheroids were then stained in toto with DRAQ5 and anti-collagen-1, followed by tissue clearing, 3D imaging, and segmentation as previously described. Initially, 130 cells within a single image were manually annotated based on the CellTracker signal. Subsequently, a support vector machine (SVM) was utilized to expand the training dataset. Based on 130 manually annotated cells, we trained a SVM with a linear kernel to identify CellTracker-positive cells, using the mean CellTracker signal intensity within the nucleus as the basis for classification. To minimize signal noise, we applied a Gaussian smoothing filter to the CellTracker signal, selecting a sigma value of 1. We evaluated the SVM model’s performance using a separate set of 142 annotated cells from a different image stack, where it achieved an accuracy of 97.9%. Subsequently, the trained SVM model was employed to analyze 5 additional image stacks, which facilitated the generation of training and testing datasets for a deep-learning classification model.

The deep-learning model’s training dataset comprised 8,081 cells from three images, while the testing dataset included 5,409 cells from 2 distinct images. The input to the deep-learning model consisted of three-channel image crops with dimensions of 48 × 64 × 64 pixels (z, y, x), centered around the nucleus. These channels represented the nuclei, collagen-1 marker, and nuclear segmentation mask, respectively. Utilizing an architecture that combines the encoder portion of a 3D U-Net segmentation model with 3 fully connected layers containing 120, 84, and 2 neurons, the model was trained from scratch. The activation functions applied were ReLU for the first two layers and Softmax for the final fully connected layer. Training utilized cross-entropy loss, with a learning rate of 1 × 10^− 4^ and the Lookahead optimizer. Data augmentation techniques—specifically flipping, rotation, scaling, contrast adjustment, embossing, perspective transformation, noise addition, and blurring—were randomly applied during training. The model was trained with a batch size of 8, continuing until no increase in validation loss was observed for 15 consecutive epochs. We selected the model weights from the epoch achieving the lowest validation loss for subsequent testing and inference, achieving a test accuracy and F-1 score of 94% and 94.1%, respectively. 3D projections and z-cuts were made using the software Vaa3D [39].

### Statistical analysis

The statistical analyses in this study were conducted using GraphPad Prism 9, ensuring all data underwent tests for normal distribution. To compare the results, we employed ordinary one-way ANOVA, incorporating Šidák’s correction for multiple comparisons for all datasets. We established a significance threshold (α) at 0.05, corresponding to a 95% confidence interval. Heatmaps were generated via GraphPad Prism, providing a streamlined visualization of the experimental data. Data normalization was conducted employing the Z-score method to ensure uniformity across datasets. This normalization involved an initial transformation of the raw data, wherein the mean value of each dataset was subtracted from individual data points to centralize the dataset around zero. Following this adjustment, the transformed data were then scaled by dividing by the standard deviation of the respective dataset. This two-step process standardizes the data, facilitating direct comparisons across different conditions and highlighting genuine experimental effects within the heatmap visualizations.

## Results

### Synergy of tissue clearing, whole mount staining, and confocal 3D microscopy enables detailed visualization of individual cell nuclei in spheroids up to 500 μm in diameter

**Fig. 2:**
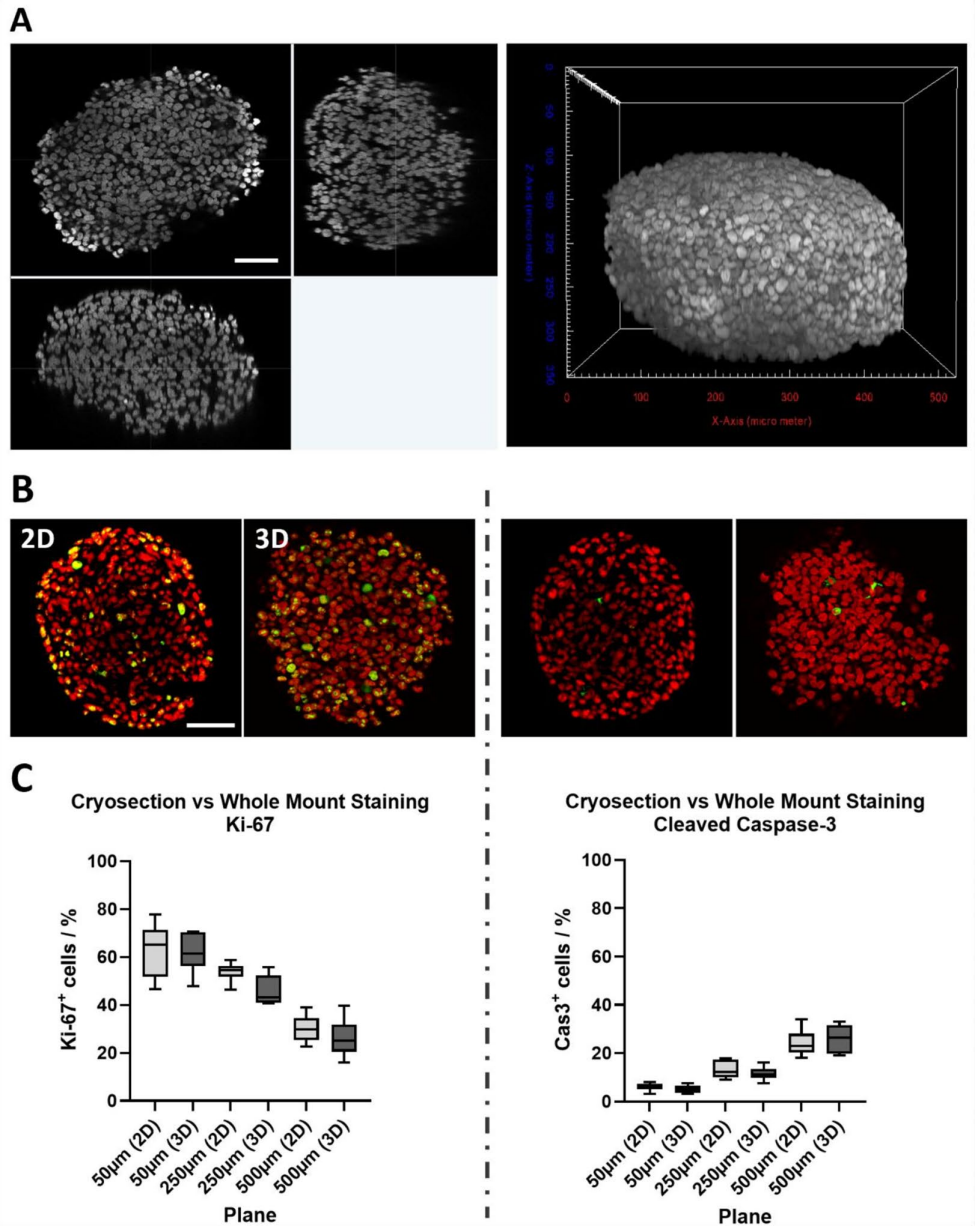
The combination of whole mount immunostaining, tissue clearing and confocal 3D-imaging allows the visualization of single cell nuclei up to a depth of 500 μm and enables comparable results of immunostaining on cryosections. KP4 spheroids of 500 μm diameter were raised. Upon fixation, spheroids were either processed as whole mounts or cryosections. All samples were immunostained against Ki-67 and Cas3, and nuclei were labeled with DRAQ5. Whole mounts were then cleared, and all samples were visualized by confocal 3D microscopy. (**A**) Orthogonal views (left panels) and corresponding volume rendering (right panel) of nuclei staining of a representative KP4 spheroid. Orthogonal views show xy- (upper left), xz- (lower left), and yz-cross-sections (upper right) through the spheroid center. Scalebar, 100 μm (**B**) Examples of single cryosections with a diameter of 250 μm (2D) and corresponding z-planes of whole mount samples (3D). Nuclei, red; immunofluorescence, green. Scalebar, 100 μm. (**C**) Box-whisker plots show the percentages of Ki-67+ (left graph) or Cleaved Caspase 3 + cells (right graph) with 3 different diameters (50, 250, and 500 μm) comparing data from cryosections (2D) and whole mount optical sections (3D). Mean ± SD (*n* = 6)

This study aimed at investigating quantitative cell-type specific drug effects at a single-cell level in mono- and co-culture spheroids composed of cancer and fibroblast cells. To achieve this, it was necessary to develop a sample preparation process that allows to reliably stain and visualize entire spheroids at a resolution sufficient to segment and count all nuclei in whole mounts of up to 500 μm in diameter. Thus, KP4-spheroids were grown up to this size, then fixed and stained for nuclei and the proliferation marker, Ki-67, or the apoptosis marker, Cleaved Caspase-3 (Cas3). Subsequently, the labeled samples were cleared and visualized by 3D confocal microscopy. As shown in Fig. 2A, depicting xy-, xz-, and yz-sections (Fig. 2A, left panels) and the corresponding volume rendering (Fig. 2A, right panel) of a representative nuclear staining, the fluorescence signals were well defined throughout the width and breadth of the image stacks.

To ascertain that impairment of dye penetration or loss of fluorescence signals due to optical light scattering or absorption in the voluminous whole mounts would not affect quantitative data assessment, the 3D-data were tested against the current gold standard, i.e., cryosections. Thus, spheroid whole mounts and cryosections were prepared side by side and the percentage of proliferative and apoptotic cells in three different optical whole mount sections taken at 50, 250, and 500 μm diameter were compared to those in cryosections taken at the same heights. Figure 2B shows exemplary images of nuclear staining (red) and Ki-67 (green, left panels) or Cas3 (green, right panels) signals on cryosections (2D) and whole mount spheroids (3D) at a diameter of 250 μm. Since KP4 cells are highly proliferative, numerous Ki-67^+^ and few Cas3^+^ cells were detected throughout the samples. The quantitative comparison presented in Fig. 2C indicates that (i) the number of proliferating cells decreased from the upper to lower planes, (ii) the relative number of apoptotic cells increased from upper to lower planes, and, importantly, (iii) the relative number of Ki-67^+^ and Cas3^+^ cells were comparable between physical and optical sections. Indeed, the relative number of Ki-67^+^ cells from physical and optical sections at depths of 50 μm (2D: 63.03 ± 4.56%; 3D: 62.31 ± 3.13%), 250 μm (2D: 54.37 ± 1.68%; 3D: 47.44 ± 1.98%), and 500 μm (2D: 30.25 ± 1.49%; 3D: 26.73 ± 1.47%) revealed the absence of significant differences (Fig. 2C, left). Similarly, there was no significant difference in the percentages of Cas3^+^ cells between optical- and cryosections with a diameter of 50 μm (2D: 6.07 ± 0.63%; 3D: 5.32 ± 0.59%), 250 μm (2D: 13.18 ± 1.70%; 3D: 11.04 ± 1.15%), and 500 μm (2D: 24.11 ± 2.26%; 3D: 26.21 ± 2.31%) (Fig. 2C, right).

**Fig. 3:**
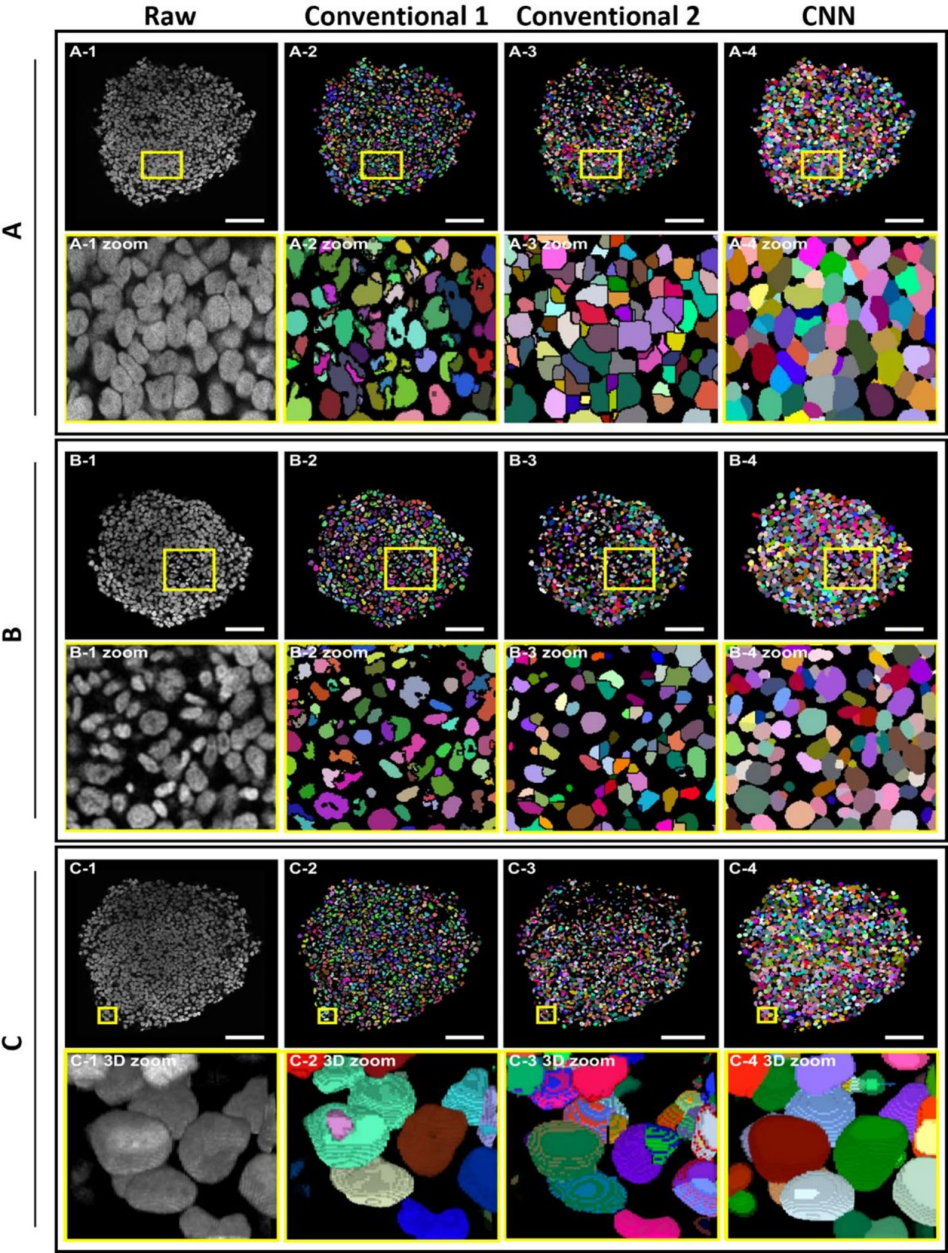
CNN-based segmentation surpasses the precision of traditional algorithms. KP4 cells were grown as spheroids up to a size of 400 μm. Then, spheroids were fixed, stained with DRAQ5, cleared, and imaged as whole mounts with a confocal microscope. Segmentation of fluorescence signals used two conventional open-source 3D-watershed-based segmentation tools (Conventional 1 and Conventional 2) and a custom-trained convolutional neural network (CNN). (**A1-C1**) Representative raw data show single optical slices in a spheroid region with a high density of nuclei (**A1**) or presence of unusual nuclear morphologies (**B1**), or a border region of the spheroid (**C1**). (**A2-C4**) Segmentation results of corresponding raw data shown in A1-C1 by algorithms, Conventional 1 (**A2-C2**), Conventional 2 (**A3-C3**), and CNN (**A4-C4**). Identified segments are depicted in different pseudocolors for better discrimination of instances. Zoom images show high-resolution images of the boxed regions in overviews on top of each zoom, except for C1-4. These show 3D-renderings of boxed regions in corresponding overviews. Scalebars: 100 μm

### CNN-Based Segmentation Outperforms Conventional Segmentation Methods Based on 3D Watershed

After the acquisition of serial whole mount 3D-image data, reliable object recognition, precise segmentation, and single-cell instance retrieval are key to any further quantitative analysis. Here, the segmentation accuracy of two conventional open-source and 3D-watershed-based segmentation tools were compared to that of a custom-trained convolutional neural network (CNN), using whole mount nuclear fluorescence 3D confocal imaging data of KP4 spheroids. When using traditional tools, the accuracy of cell nuclei instance detection accuracy was particularly low in regions with high cell density and increasing fluorescence signal overlaps (compare high-density-area in Fig. 3A1-3 and low-density area in Fig. 3B1-3). This resulted in significant nuclear under- and over-segmentation. Also, the shapes of nuclei were often not reliably detected by conventional algorithms, leading to straight edges or holes in nuclear regions of interest. Conversely, the CNN-based method led to a substantially higher accuracy in spotting overlapping nuclei in dense regions and in retrieving nuclear morphology (Fig. 3A4 and Fig. 3B4). Additionally, the CNN identified a higher number of darker nuclei, most of which were missed by the two conventional tools. Finally, also the 3D-morphology, which is essential for an accurate determination of nuclear dimensions, was recognized more reliably by the CNN (compare Fig. 3C4 to Fig. 3C2-3). A quantitative comparison of the segmentation performance was done using previously introduced segmentation (SEG) and detection (DET) scores [36]. The two conventional segmentation methods achieved a SEG of 0.015 (conventional method 1) and 0.019 (conventional method 2), while the CNN-based segmentation was significantly better with a SEG score of 0.532. Similarly, the conventional segmentation algorithms yielded DET scores (0.59 for conventional method 1 and 0.61 for conventional method 2, respectively) that were significantly lower than the 0.893 reached by the CNN. In summary, the CNN-based algorithm segmented the 3D-data sets used in this work much more precisely than the conventional methods and was therefore used for all further analyses in this work.

### Cytostatic treatments primarily affect cancer cells and lead to massive changes in spheroid morphologies

**Fig. 4:**
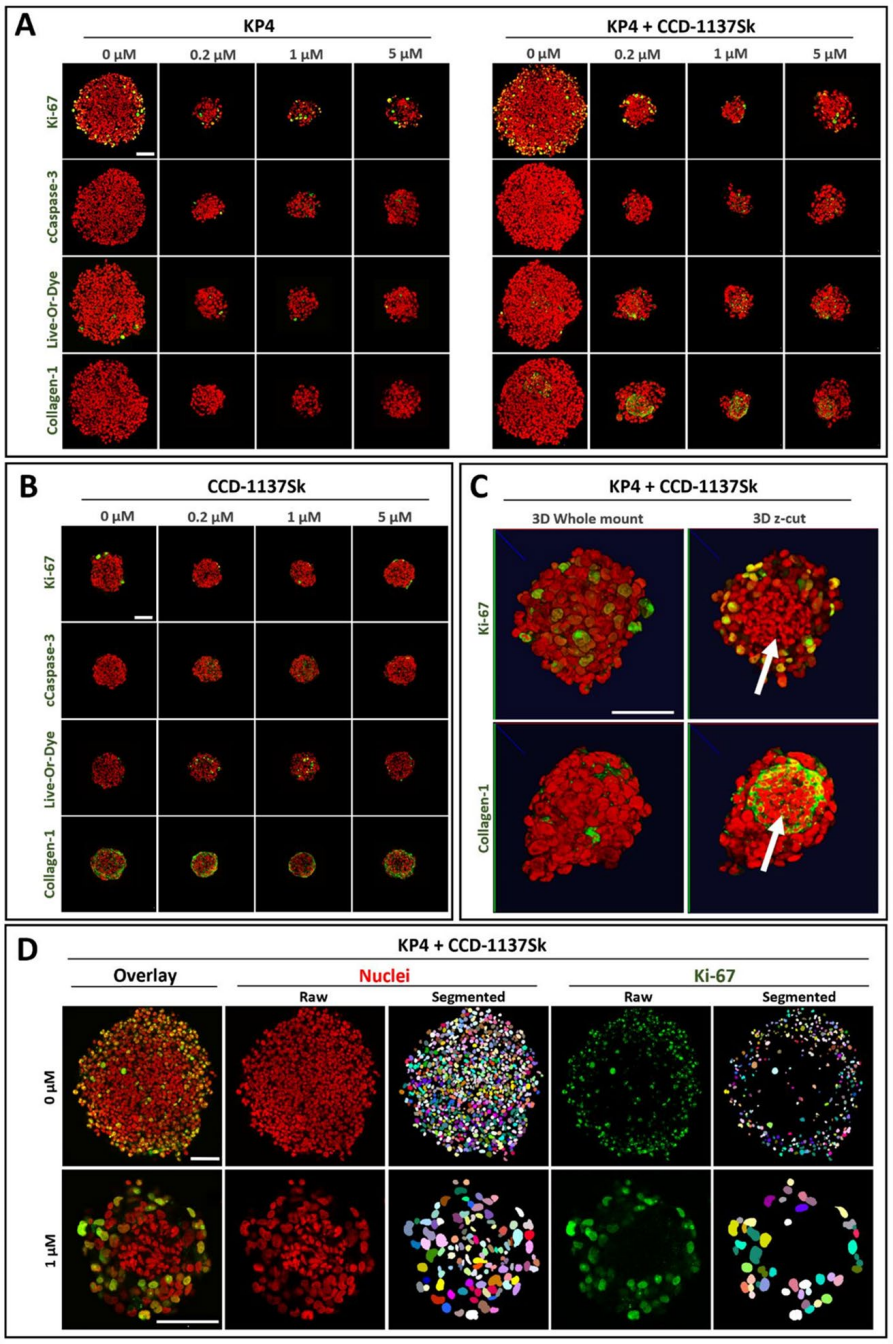
Paclitaxel treatment exhibits differential effects on KP4 cancer cells and CCD-1137Sk fibroblasts. KP4 tumor cells and CCD-1137Sk fibroblasts were seeded into 96-well ULA plates in mono- and co-culture and treated with paclitaxel after three days in culture for 96 h at four different concentrations (0, 0.2, 1, and 5 µM). Then, spheroid whole mounts were fixed, cleared and stained with fluorescence markers to detect proliferation (Ki-67), apoptosis (Cleaved Caspase-3), necrosis (Live-or-Dye), fibroblasts (collagen-1), and nuclei (DRAQ5). Whole mount 3D confocal microscopy and CNN-based 3D-image segmentation were performed. All scalebars, 100 μm. (**A-B**) Representative micrographs showing single optical sections through spheroids in KP4 mono- (**A**,** left panels**) or co-culture (**A**,** right panels**) or CCD-1137Sk mono-culture (**B**) at the largest spheroid circumference with indicated fluorescence markers. Nuclei (red) and marker signals (green) are shown as overlays. (**C**) Representative 3D-volume projections of co-culture spheroids stained with DRAQ5 (red), Ki-67 (green, top), and collagen-1 (green, bottom) treated with 1 µM paclitaxel. The right panels show optical z-cuts at the spheroid center. They highlight the localization of the fibroblasts, which are characterized by small, elongated nuclei (indicated by white arrows) and a high amount of collagen-1. (**D**) Representative segmentations of nuclei and Ki-67^+^ cells, as indicated from KP4 cocultures treated with 0 µM (top row) or 1 µM paclitaxel (bottom row)

To evaluate the effects of the cytostatic drugs on the different culture conditions, KP4 tumor cells and CCD-1137Sk fibroblasts were seeded into 96-well ULA plates in mono- and co-culture. After three days in culture, the resulting spheroids were incubated with either paclitaxel or doxorubicin at four different concentrations (0, 0.2, 1, and 5 µM) for 96 and 144 h. Then, spheroids were fixed, cleared, and stained with fluorescence markers to detect proliferative (Ki-67), apoptotic (Cas3), and necrotic (Live-or-Dye) cells. Furthermore, collagen-1 was used as a marker for fibroblasts, and nuclei were labeled with DRAQ5. The cytotoxic effects of the chemotherapeutics were visualized on a single-cell level by 3D confocal microscopy. Representative micrographs of single optical sections from whole mounts of spheroids in mono- and co-culture, captured at their largest circumference, provided a first qualitative summary of the results. Indeed, for both drugs, we observed a concentration-dependent decrease in the diameters of the spheroids. Notably, after 144 h, the samples exhibited more pronounced effects, aligning with the trend noted at 96 h (Fig. 4A-B, Fig. S1).

However, while the sizes of KP4 spheroids treated with paclitaxel, both in mono- and co-culture, were massively reduced at the lowest drug concentration and remained constantly small at higher drug levels (Fig. 4A, Fig. S1A), CCD-1137Sk spheroids showed only a moderate size reduction under all treatment conditions (Fig. 4B). In contrast, mono- and co-cultured KP4 spheroids treated with doxorubicin showed a significant, concentration-dependent reduction in size. This size reduction was also observed in fibroblast spheroids, albeit to a lesser extent, and became only clearly pronounced at the highest drug concentration (see Fig. S1A and B). This suggested that CCD-1137Sk cells were less affected by the cytostatic drugs than the KP4 cells. That assumption was corroborated by the collagen-1 staining in co-culture spheroids, which displayed a relatively stable appearance in the core of control and treated spheroids (Fig. 4A, right panels; Fig. S1A, right panels). Notably, fibroblasts showed very little proliferation (Fig. 4B, Fig. S1B) and their nuclei were visibly smaller than those of KP4 cells (see arrows in Fig. 4C). Thus, the different mono- and co-culture conditions as well as the drug treatments led to significant changes in overall spheroid appearance, presence of markers, and nuclear morphologies (e.g., interphase, mitotic, apoptotic, small, elongated fibroblast nuclei vs. round-elliptic large KP4 nuclei). Nonetheless, the CNN-based segmentation reliably identified nuclei and marker-positive cells (Fig. 4D).

**Fig. 5:**
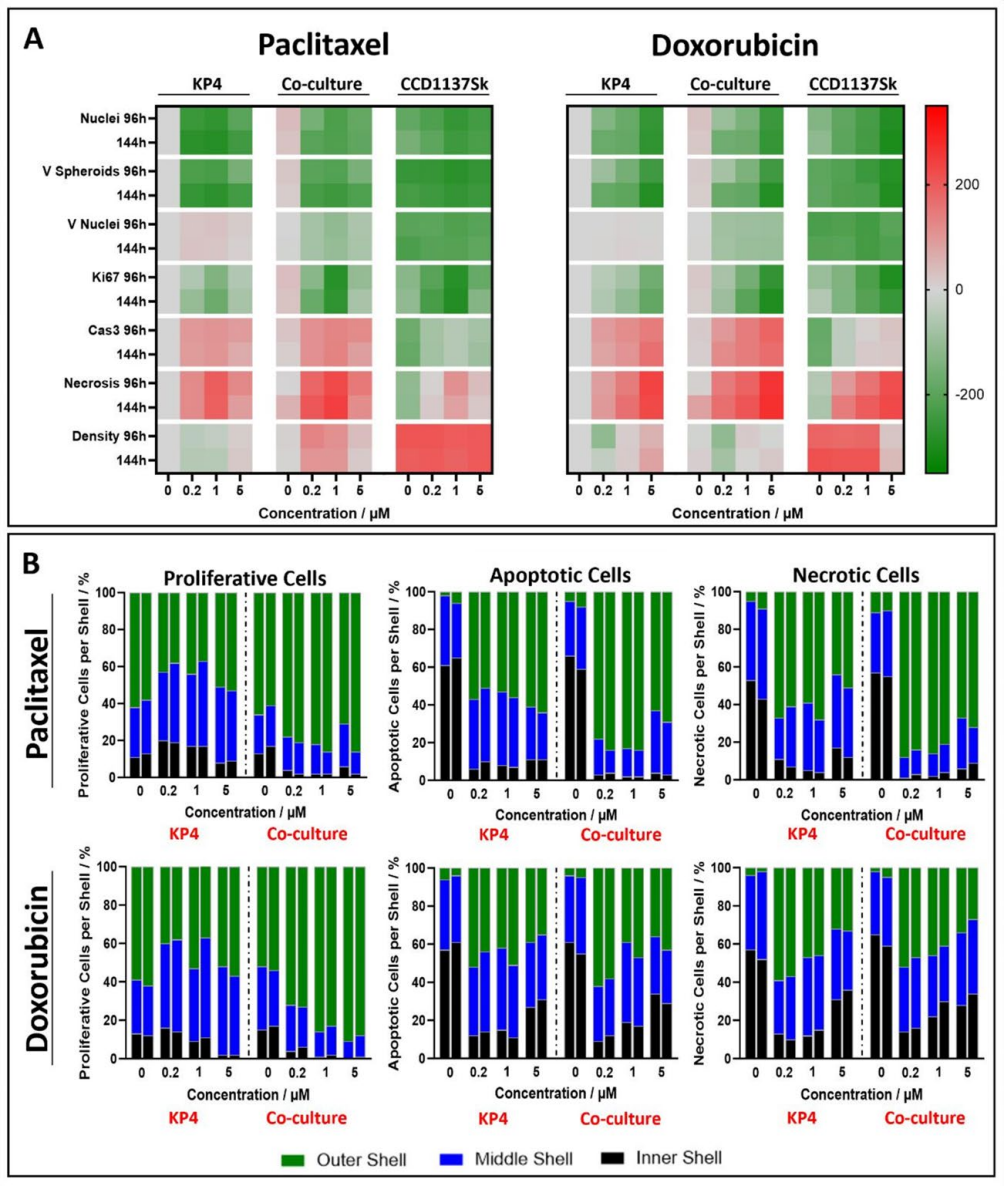
In co-culture with CCD-1137Sk fibroblasts, KP4 spheroids exhibit a differential basic activity and response to cytostatic treatment. KP4 tumor cells and CCD-1137Sk fibroblasts were seeded into 96-well ULA plates in mono- and co-culture and treated with paclitaxel or doxorubicin after three days in culture for 96–144 h at four different concentrations (0, 0.2, 1, and 5 µM). Then, spheroid whole mounts were fixed, cleared and stained with fluorescence markers to detect proliferation (Ki-67), apoptosis (Cas3), necrosis (Live-or-Dye), fibroblasts (collagen-1), and nuclei (DRAQ5). Whole mount 3D confocal microscopy and CNN-based 3D-image segmentation was performed. (**A**) The heatmaps show z-scored comparisons between all different culture conditions (KP4 mono-cultures (KP4); KP4 + CCD-1137Sk co-cultures (Co-culture); CCD-1137Sk mono-cultures (CCD1137Sk)), drug incubation concentrations and times for seven different characteristics, i.e., number of nuclei per spheroid (Nuclei), volumes of spheroids (V Spheroids), volumes of nuclei (V Nuclei), percentage of Ki-67^+^ cells (Ki67), percentage of Cas3^+^ cells (Cas3), percentage of Live-or-Dye^+^ cells (Necrosis), and number of nuclei per µm^3^ (Density). Per each column, data were normalized to the corresponding value determined for KP4, 0 µM drug. Data show z-scored increase (red) or decrease (green) of values. See color scalebar for reference. (**B**) 3D-shell analysis was performed on spheroid confocal stacks divided into outer (green bars) and middle shell (blue bars), and spheroid core (black bars) to investigate the spatial distribution of proliferating, apoptotic, and necrotic cells. The stacked bars show the percentage distribution of the individual markers from six spheroids for each condition. Two neighboring bars indicate values after 96 h (left columns) or 144 h (right columns) of drug treatment

### Co-culture spheroids of KP4 and CCD-1137Sk cells show enhanced proliferation and reduced susceptibility to cytostatic treatment

The high microscopic quality of the immunofluorescence-stained 3D whole mounts, combined with a precise segmentation, facilitated accurate quantification of the cytotoxic effects of paclitaxel and doxorubicin. This quantification was achieved through the analysis of a series of markers and morphological features extracted from the 3D confocal image data stacks, following CNN-based 3D-image segmentation. The utilized markers included DRAQ5 for counting nuclei and measuring nuclear volumes, Ki-67 for identifying proliferating cells, Cas3 for detecting apoptotic cells, and Live-or-Dye for identifying necrotic cells. Additionally, the volumes of the spheroids and the density of nuclei within the spheroids were assessed as morphological features. While Figures S2-S3 and Tables S1-S2 summarize all data graphically and numerically, Fig. 5A shows heatmaps as a quick reference for the major effects that could be observed for both cytostatic substances. In the heatmaps, all values were z-score normalized (grey shading) to those for KP4 spheroids in the absence of cytostatic.

Briefly, the principal observations were as follows: (i) Upon all cytostatic incubations, KP4 mono-culture and KP4 + CCD-1137Sk co-culture spheroids showed a massive decrease in the number of nuclei, spheroid volume, and cell proliferation as well as an increase in apoptosis, and necrosis. (ii) While for paclitaxel the described effects were maximal at 1 µM concentration, doxorubicin effects further increased at 5 µM concentration. (iii) In CCD-1137Sk mono-culture spheroids, these effects were less pronounced, but also visible. (iv) The average volume of nuclei remained stable in untreated mono-cultures and co-cultures but increased in 0.2 and 1 µM paclitaxel-treated mono-cultures. The co-cultures, however, exhibited a decrease in average nuclei volume with increasing drug concentration. For doxorubicin, this effect was particularly evident in co-cultures across all concentrations. (v) KP4 mono-cultures treated with 0.2 and 1 µM paclitaxel showed a significant decrease in spheroid density at both time points, while co-culture groups exhibited a significant increase across all treated groups, except for 5 µM at 144 h. Treatment with 0.2 µM doxorubicin decreased spheroid density in both KP4 mono- and co-cultures, while 5 µM resulted in a significant increase in spheroid density in KP4 mono-cultures. The densities in CCD-1137Sk mono-cultures were mainly unaffected by treatment with both substances; except for doxorubicin groups treated with 5 µM, which exhibited a significant reduction. (vi) In a direct comparison between KP4 mono-culture and KP4 + CCD-1137Sk co-culture spheroids, the latter showed higher nuclei counts, spheroid sizes, and proliferative and necrotic activity in the absence of chemotherapeutics. Upon treatment, loss of nuclei and spheroid volume were less pronounced in the co-cultures, although the reduction in proliferation and the increase in necrosis were both enhanced. (vii) Co-culture spheroids showed a reduced average nuclear volume upon treatment. In summary, these data suggested that CCD-1137Sk fibroblasts responded less to the treatments than KP4 cells and that co-cultures were less susceptible to drug incubation than KP4 mono-culture spheroids.

In addition to the general quantification of molecular and morphological features, we conducted a 3D shell analysis to assess the spatial distribution of proliferation, apoptosis, and necrosis across three segments of the spheroids: the spheroid rim, middle ring, and core (Fig. 5B). The major findings of this analysis were: (i) Without treatment, the distribution of all markers was similar between mono- and co-cultures, showing about 90% of proliferating cells in the middle and outer spheroid regions, while 50–60% of apoptotic and necrotic cells were located in the spheroid cores. (ii) Upon paclitaxel and doxorubicin treatment, the number of proliferating cells decreased in the cores of co-culture spheroids, while it was unaffected in mono-cultures (except for doxorubicin at 5 µM). (iii) Both paclitaxel and doxorubicin led to increased apoptosis and necrosis in the outer and middle rings; this effect was more pronounced with paclitaxel.

**Fig. 6:**
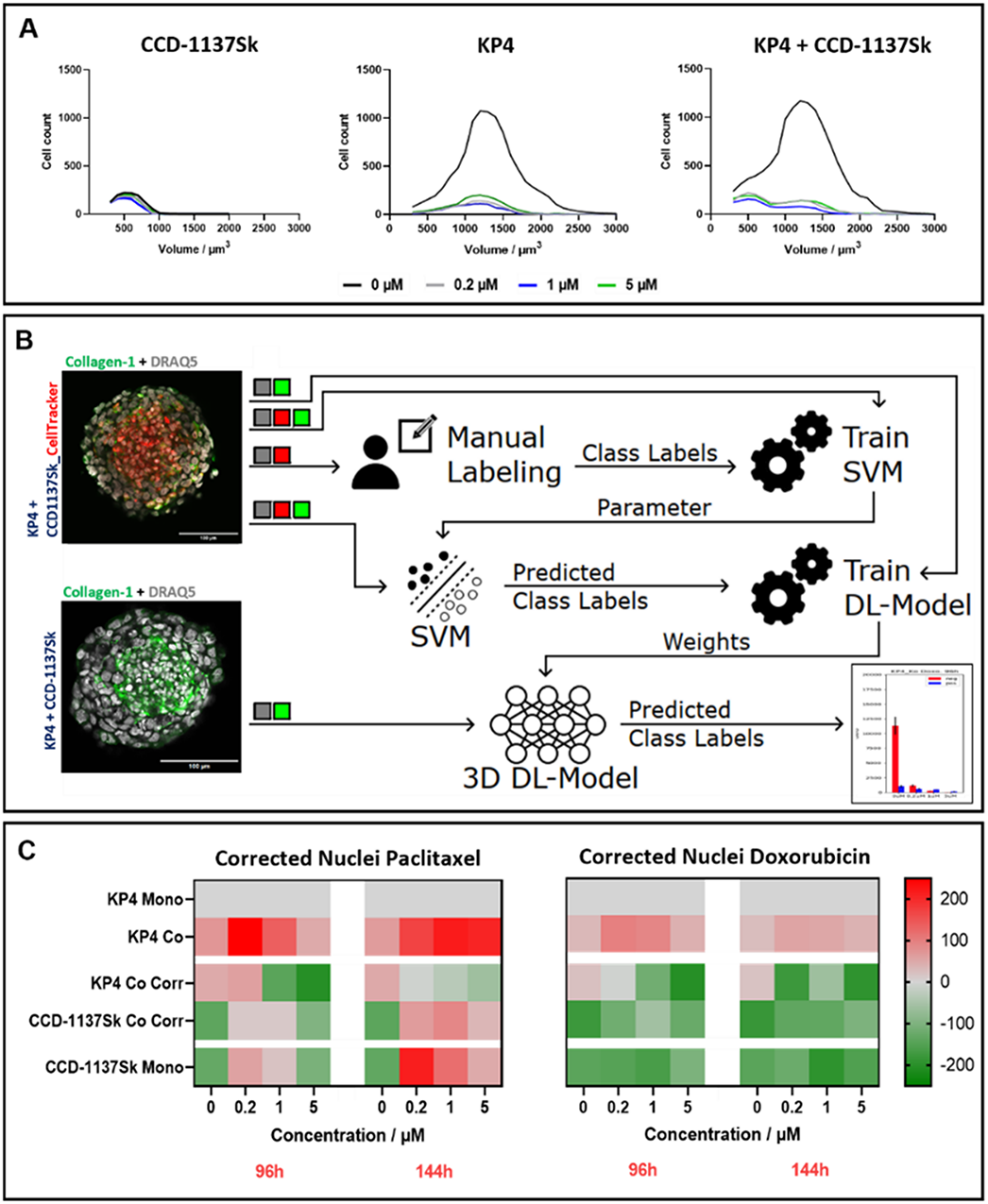
Cell-type-specific analysis indicates enhanced KP4 cell growth in co-cultures and higher susceptibility of KP4 cells to cytostatics. KP4 tumor cells and CCD-1137Sk fibroblasts were seeded into 96-well ULA plates in mono- and co-culture and treated with paclitaxel or doxorubicin after three days in culture for 96–144 h at four different concentrations (0, 0.2, 1, and 5 µM). Then, spheroid whole mounts were fixed, cleared and stained with fluorescence markers to detect nuclei (DRAQ5) (**A**-**C**) and collagen-1 (**B**). In addition, for some experiments, CCD-1137Sk cells were labeled with CellTracker prior to spheroid formation (**B**). Whole mount 3D confocal microscopy and CNN-based 3D-image segmentation were performed. (**A**) Frequency distribution of nuclear volumes from CCD-1137Sk and KP4 mono- and co-cultures, treated with four different concentrations of paclitaxel for 96 h, as indicated. (**B**) Schematic drawing to illustrate the development of a 3D DL-Model for automated segregation of nuclei according to cell type. To distinguish between KP4 and CCD-1137Sk cell nuclei in co-cultures, co-culture spheroids were prepared either with or without CellTracker-labeled fibroblasts, stained with anti-collagen-1 and DRAQ5, and imaged with 3D confocal microscopy. The images were used to train a support vector machine (SVM) including different channel inputs. The SVM-predictions trained a 3D DL-Model to identify fibroblast class labels from specific image channels. (**C**) Z-score normalized heatmaps show corrected nuclei numbers from co-cultures treated either with paclitaxel or doxorubicin at four different concentrations for 96 and 144 h of three biological replicates, each with six technical replicates. Corrected values from co-cultures display the quantity of KP4 tumor cells (KP4 Co Corr) and CCD1137Sk fibroblasts (CCD-1137Sk Co Corr) individually within the co-cultures (KP4 Co) and were calculated by subtracting predicted fibroblast nuclei from total nuclei numbers of the corresponding co-culture group. Per each column, data were normalized to the corresponding value determined for KP4 Mono, 0 µM drug. Data show z-scored increase (red) or decrease (green) of values. See color-scalebar for reference

### Cell-type specific analysis reveals particular susceptibility of KP4 cells to cytostatic drugs

The previous analyses had suggested that co-culture spheroids, composed of KP4 cancer and CCD-1137Sk fibroblast cells, were differently susceptible to paclitaxel and doxorubicin than KP4 mono-culture spheroids. Earlier, it was noted that nuclei of fibroblasts were smaller and more elongated than those of KP4 cells (see, e.g., Fig. 4C). Thus, to better understand potential cell-type specific effects, we analyzed the size distributions of nuclear volumes in untreated and treated mono- and co-culture spheroids. This yielded very clear differences (Fig. 6A). While the nuclei of CCD-1137Sk mono-culture spheroids showed a single maximum of approximately 500 µm^3^, nuclear volumes of untreated KP4 mono-culture spheroids exhibited a binomial distribution with a peak at 1,200 µm^3^. Notably, also untreated KP4 + CCD-1137Sk co-culture spheroids showed a roughly binomial curve centered at 1,200 µm^3^. But in addition, there was a shoulder at 500 µm^3^, suggesting that this reflected the mix of nuclei from both cell types. Now, when looking at the distributions in the presence of paclitaxel, the curves for fibroblast mono-cultures were almost unaltered and those of KP4 mono-cultures were basically just flattened, when compared to untreated conditions. Conversely, the distribution curves of treated co-cultures showed a switch of peak and shoulder, i.e., now the maximum was found at 500 µm^3^ and a shoulder at 1,200 µm^3^. This suggested that (i) the nuclei of the two cell lines might be discriminated by virtue of their volume and (ii) upon cytostatic treatment, there was a preferential loss of KP4 nuclei in co-culture spheroids.

To follow up on these hypotheses, we developed a supervised 3D deep-learning (DL) model (Fig. 6B) to discriminate nuclei of KP4 from those of CCD-1137Sk cells in co-culture spheroids. To that end, co-culture spheroids were generated with unmarked KP4 cells and CellTracker-labeled fibroblasts. Whole mounts were fixed, stained with DRAQ5 and anti-collagen-1 antibody, and then imaged using 3D confocal microscopy. These data were first employed to train a support vector machine, which received a combination of fluorescent nuclear, CellTracker, and collagen-1 channels as input 1 and image stacks with only nuclear and CellTracker channels, where fibroblasts were manually classified as input 2. From the resulting decision parameters and the input of image stacks with all three channels, the SVM was enabled to predict class labels. These class labels were employed to train a 3D DL-model which should predict fibroblast class labels from only nuclear and collagen-1 channel image stacks (Fig. 6B).

Application of the 3D DL-model to the cytostatic treatment data as discussed in the description to Fig. 5 led to corrected nuclei numbers (Fig. 6C, Fig.S4, and Table S3). Figure 6C shows heatmaps as a quick reference for the major effects that could be observed. In those heatmaps, all entries were z-score normalized to the corresponding KP4 mono-culture condition. The most important findings of this analysis were as follows: (i) In the uncorrected mode (see Fig. 6C, KP4 Mono and KP4 Co), co-cultures showed under all drug incubations higher nuclei counts than KP4 mono-cultures. As documented in Table S3, there were about 42% more nuclei. Upon DL-mediated assignment of nuclei in co-culture spheroids to either KP4 or CCD-1137Sk cells (see Fig. 6C, KP4 Co Corr and CCD-1137Sk Co Corr), the picture was more differentiated: (ii) For all conditions in the absence of cytostatic, there were more KP4 nuclei in the co-cultures than in the KP4 mono-cultures (about 30%, see Table S3); (iii) conversely, the number of CCD-1137Sk nuclei was inferior to the total number of nuclei in untreated KP4 mono-cultures (about 11%, see Table S3). (iv) In the presence of 0.2 µM and 1 µM of paclitaxel (and at 144 h also for 5 µM of the drug), the number of CCD-1137Sk nuclei in co-cultures exceeded that of KP4 nuclei in KP4 mono-cultures, while the number of KP4 nuclei in the treated co-cultures diminished with increasing paclitaxel concentrations. (vi) In the presence of doxorubicin, both KP4 and CCD-1137Sk nuclei in co-cultures were less in number than the nuclei in treated KP4 mono-cultures, but in sum, cancer cell and fibroblast nuclei exceeded the amount of KP4 mono-culture spheroids. (vii) Finally, in the presence of paclitaxel at 144 h, nuclei in treated fibroblast mono-cultures exceeded the numbers of nuclei in KP4 mono-cultures, while they were inferior upon treatment with doxorubicin.

## Discussion

Stromal cells are known to markedly affect metabolism, growth, and drug resistance of cancer cells [40–44]. In this context, 3D cell cultures, such as tumor spheroids, xenografts, and tumor organoids, have been increasingly used to address drug efficacy [45–48]. To identify stromal-induced modulation of neoplastic cells, co-culture spheroid models were commonly used. To discriminate between the cell types, labeling with GFP, dyes, or cell-type specific surface antigen antibodies followed by cell sorting approaches were often deployed [49–52]. Alternatively, stromal effects were indirectly deduced from general comparison of the whole mono- and co-culture parameters, such as spheroid size upon treatment or relative expression of genes or proteins [53–55]. As valuable as these approaches were for detecting stromal-induced effects on cancer cell behavior, they mostly came with a dissociation of the 3D-cultures and therefore the spatial localization of drug effects within the cell aggregate was lost. Alternatively, 3D-culture integrity was maintained, but then the stromal-effects were only assigned to the spheroids as a whole [56–60]. In other terms, a non-destructive means to enable the analysis of cell-type specific effects on the single-cell level in 3D cell cultures remained elusive. Here, we describe a pipeline that combines optical tissue clearing, whole mount staining, and confocal microscopy with a CNN-based segmentation algorithm and a 3D deep-learning model for a reliable discrimination of cancer and fibroblast cells in large co-culture spheroids. An additional benefit of our method is that fibroblast labeling using a marker panel with multiple antibodies is not required for characterization under co-culture conditions.

However, to achieve this, high segmentation accuracy is crucial. Therefore, we used a deep-learning-based segmentation method that has demonstrated robust performance under heterogeneous conditions, as evidenced by its success in the CellTracking Challenge in contrast to traditional segmentation algorithms [36]. This method is particularly effective at handling variability in cell morphology and high cell density, which are common challenges in biological imaging. The primary reason for lower accuracy in high-density regions with overlapping fluorescent signals when using conventional tools is their limited ability to distinguish closely packed nuclei [61]. Conventional segmentation algorithms often struggle to separate touching or overlapping nuclei, leading to errors in instance detection. These methods typically rely on predefined thresholds or simple morphological operations, which are often insufficient in complex environments. To address these challenges, we not only used an established deep-learning segmentation method but also incorporated synthetic training data, specifically designed to include scenarios with high nuclei density and signal overlap, as described in the Materials and Methods section. The use of synthetic training data allowed us to simulate and train the model on conditions that closely mimic the challenging aspects of our experimental data. As a result, we improved the model’s ability to discern individual nuclei even in densely populated regions, thereby increasing segmentation accuracy.

Application of the pipeline to KP4 lung cancer and CCD-1137Sk fibroblast co-culture spheroids under cytostatic treatment revealed that in untreated co-cultures the KP4 cells had a growth advantage over KP4 mono-culture spheroids but were also more susceptible to cytostatic drugs than the CCD-1137Sk fibroblasts. These findings align with prior studies showing that co-cultures with fibroblasts can promote tumor cell proliferation via the release of numerous factors, such as fibroblast growth factor (FGF), hepatocyte growth factor (HGF), and transforming growth factor-beta (TGF-β), that promote tumor cell proliferation [62–65]. Several studies illustrated how fibroblast-derived cytokines and extracellular matrix components can enhance tumor growth through paracrine signaling and matrix stiffening, which activate mechanotransduction pathways in tumor cells [66, 67]. Additionally, fibroblasts help remodel the ECM, influencing tumor cell adhesion and migration, contributing to an environment conducive to tumor expansion [68].

Moreover, it has also been demonstrated that the sensitivity of fibroblasts to chemotherapeutic agents such as doxorubicin and paclitaxel varies and is influenced by several factors including the proliferation rate, the origin of fibroblasts, the presence of other cellular factors, drug concentration, and the genetic makeup of the cells [69–73]. It is expected that a careful selection of cell-type intrinsic characteristics, such as nuclear volume or marker expression, in combination with the presented pipeline, will be useful to dissect cell-type specific effects also in more complex co-culture spheroids or other 3D-cultures at a single-cell resolution.

In our study, treated cocultures consistently exhibited an increased number of cell nuclei in the absence of drugs as well as across most concentrations of both drugs, when compared to mono-cultures. This suggests that the presence of fibroblasts led to a growth advantage of KP-4 cells as well as to a potential increase in chemo-resistance. Both findings are consistent with previous studies suggesting that the tumor microenvironment, including the presence of cancer-associated fibroblasts (CAFs), may contribute to enhanced cancer cell growth [12] but also to therapeutic resistance. The phenomenon of chemotherapeutic resistance in tumor cells induced by co-cultured CAFs is a complex and multifaceted issue [74–82]. In pancreatic ductal adenocarcinoma (PDAC), CAFs contribute particularly to chemotherapeutic resistance through various mechanisms such as secretion of growth factors and cytokines, inhibition of immune cell infiltration, activation of STAT3 signaling, upregulation of CXCR2, exosome secretion, microRNA signaling, and metabolic reprogramming [83–90]. However, recent findings extend beyond the established role of CAFs in mediating chemotherapeutic resistance. Several studies have shown that non-tumorigenic fibroblasts can also induce resistance to various chemotherapeutic agents, when co-cultured with tumor cells [13, 51, 91–96]. Conversely, although we found significantly higher cell counts in most co-culture groups in our study, we observed a decrease in proliferating and an increase of apoptotic and necrotic cells in cocultures, accompanied by a shift in average nuclear volumes towards smaller sizes, and a higher degree of spheroid compactness, resembling those of fibroblasts, rather than tumor cells. Surprisingly, upon classification and removal of fibroblasts, the number of tumor cells was significantly lower in most co-culture groups, indicating an increased sensitivity of the tumor cells to the cytostatic drugs in the heterotypic spheroids. This observation indicates that although the total number of cells in most treated cocultures was higher than in corresponding mono-cultures, there was a relative decrease in tumor cells, while fibroblasts were more resistant, resulting in a fibroblast-dominated environment. Thus, while the initial overall cell count analysis suggested chemoprotective effects induced by fibroblasts, the more detailed analysis by cell type revealed that the increased cell counts in co-cultures were due to the predominance of the less sensitive fibroblasts. Interestingly, such increased drug sensitivity is in line with other studies that have shown that co-culturing fibroblasts with tumor cells enhanced sensitivity to chemotherapeutic agents. Indeed, it has been demonstrated that the drug-sensitizing influence can be variable among tumor cell lines rather than between tumor entities [97–104]. Furthermore, it can also depend on the anatomical origin of fibroblasts [105–107]. In addition, the use of medium supernatant derived from fibroblasts may also trigger sensitizing effects [108–111]. For example, Morales et al. showed that supernatant from healthy fibroblasts potentiated the efficiency of drugs on melanoma cells, while those from CAFs tended to increase cancer cell survival [112]. Other studies indicated that fibroblasts may also cause drug-dependent sensitization effects on tumor cells. For instance, coculturing pancreatic tumor cells with fibroblasts increased their sensitivity to gemcitabine, but not to paclitaxel and SN38 [113]. Finally, Majety et al. highlighted varied response of different drugs across diverse co-culture tumor models [102].

Recent studies have shown that fibroblasts can enhance tumor cell sensitivity to chemotherapeutics through various mechanisms. One such mechanism may be nutrient competition: fibroblasts co-cultured with tumor cells may compete for nutrients and oxygen, leading to metabolic stress in tumor cells and increase their vulnerability to chemotherapy [114, 115]. Paracrine signaling is another key mechanism by which fibroblasts influence tumor drug response. Cancer-associated fibroblasts (CAFs) secrete cytokines, including interleukins and extracellular vesicles that can enhance apoptotic signaling within tumor cells, promoting drug sensitivity. For instance, certain interleukin-related signals released by CAFs stimulate apoptosis-associated pathways in tumor cells, thereby increasing their responsiveness to chemotherapy [115, 116]. Additionally, exosomes from fibroblasts can carry microRNAs that modulate tumor cell drug responses, adding another layer of drug sensitization [117–119]. Furthermore, metabolic reprogramming may also play a role. Fibroblasts can alter the metabolic state of nearby tumor cells, often elevating oxidative stress and impacting drug efficacy. Findings by Li et al. [120] demonstrate that fibroblasts can increase reactive oxygen species (ROS) levels in tumor cells, making them more sensitive to ROS-dependent drugs like doxorubicin. In addition, increased tumor cell proliferation in co-culture with fibroblasts could lead to enhanced drug sensitivity, as cytostatic agents often target actively dividing cells. Tumor cells with higher proliferation rates are generally more susceptible to chemotherapy because more cells are in phases of the cell cycle where cytostatic agents are most effective, thus a higher proportion of proliferating tumor cells can lead to a stronger cytotoxic effect due to the increased pool of drug-sensitive cells [121]. These interconnected mechanisms underscore the complex role of fibroblasts in modulating tumor cell growth and enhancing drug responsiveness in co-culture settings. A limitation of our study may be, that we used CCD-1137Sk foreskin fibroblasts instead of cancer-associated fibroblasts (CAFs), and it may be a relevant point that different fibroblast types might yield variable responses in tumor cell behavior due to the intrinsic heterogeneity of fibroblast populations. However, numerous publications indicate that not only cancer-associated fibroblasts (CAFs), but also normal fibroblasts can significantly influence the behavior of tumor cells in co-culture models. For example, normal fibroblasts have been shown to transform into CAF-like cells when exposed to the tumor microenvironment, affecting tumor progression through similar mechanisms as primary CAFs [122]. Additionally, fibroblasts derived from different anatomical origins, such as dermal or pancreatic fibroblasts, may respond differently in co-cultures due to varying gene expression profiles, cytokine secretion patterns, and ECM remodeling abilities [123]. Future work will address this limitation by incorporating primary CAFs and other fibroblast types from various tissue origins to better understand how these cells might differently influence tumor growth, drug sensitivity, and resistance mechanisms. By expanding our model in this way, we aim to further elucidate the impact of fibroblast heterogeneity on co-culture dynamics, providing more clinically relevant data for diverse tumor microenvironments. Furthermore, we were able to confirm that traditional luminescence-based drug response results could be successfully recapitulated using our novel 3D Drug Testing Pipeline. For instance, we observed, that while paclitaxel demonstrated significant efficacy in killing cancer cells at a concentration of 1 µM, its effectiveness was notably reduced at 5 µM. This counterintuitive observation is known in literature and was attributed to cell-type specific activation of ATP-Binding Cassette (ABC) transporters in cancer cells, e.g., such as overexpression of P-glycoprotein 1 (ABCB1/P-gp), leading to paclitaxel resistance at higher concentrations [124–127]. Further ABC family members are also known to play a pivotal role in the development of drug resistance [128–134].

To date, numerous studies analyzed in 3D the influence of fibroblast co-cultivation on the efficacy of tumor drug treatments at the single-cell level using machine-learning-based methods [135–140]. While these studies gave valuable insights into the complex interactions between tumor cells and their microenvironment, they typically required the fibroblasts to be pre-labeled with fluorescence dyes for subsequent tracking. An alternative method involved post-cultivation staining of specific cell markers with fluorescence-conjugated antibodies. However, fibroblasts in the tumor environment are known for their remarkable plasticity, with their marker expressions varying depending on their origin, degree of differentiation, and specific phenotype. Consequently, characterizing fibroblasts often necessitates a panel of different markers [141–145]. A significant advancement of our study is the introduction of a simplified characterization of fibroblasts using an SVM-based approach. This method allows for efficient classification using just collagen-1 and a nuclear dye. By minimizing the need for multiple markers, our approach not only reduces the time and consumption of sample material but also enhances efficiency. This is particularly beneficial for the compatibility of our platform with primary patient samples, which is crucial for personalized treatment approaches.

Furthermore, various open-source 3D image analysis tools based on machine learning have been published for the analysis of 3D cell cultures [61, 146–154]. However, most of these image analysis packages are specialized to answer specific questions, which limits their utility in broader research contexts or are restricted to a predefined set of metrics for quantifying morphological features [155–162]. A further notable drawback is the lack of interoperability with segmentation masks generated by other deep-learning networks such as Cellpose [163] or Stardist [164]. Moreover, generating specific metrics often requires the installation and use of various software packages, making the process complex and time-consuming [165–168]. These limitations highlight the need for the development of more versatile, user-friendly, and comprehensive 3D analysis tool that can quantify a wider range of cell-type specific morphological features of 3D datasets at the single-cell level. The Python script we developed is at the centerpiece of the 3D downstream analysis. It has a user-friendly graphical user interface that enables the convenient selection of markers and segmentation masks for quantification. This selection is used to calculate various metrics, including spheroid density, spheroid volumes, distance to the hull as well as to the center, and the spatial distribution of markers through a novel 3D-shell analysis. This analysis is a core feature of our script. The calculation of the shells is not based on simple spherical approximations, but uses the actual distances to the spheroid hull, which leads to more consistent subdivisions in terms of nutrient gradients. In addition to the spatial analysis of nuclei and marker distribution in 3D, it provides further insights like mean nuclear volumes and nuclei class ratios for each shell, enhancing the overall depth and comprehensiveness of our approach. A further notable feature is its ability to accept the segmentation outputs of any neural network. This flexibility represents a significant improvement over most other software packages. Furthermore, our script allows for the determination of intensities at the single-cell level and extracts various morphological metrics using the `regionprops` function from the `scikit-image package`. To the best of our knowledge, our tool is the first of its kind to combine a wide range of metrics into a single software package. It is particularly user-friendly, allowing for fully automated batch processing without requiring additional user input. This feature makes it a valuable tool for researchers who wish to perform complex 3D image analyses without having to delve into the technical details of image processing.

## Conclusions

The results of this study highlight the critical importance of conducting cell-type-specific evaluations of drug efficacy studies at the single-cell level, as this can significantly influence the outcome of the drug response analysis. The analysis model proposed in this study is novel in several key aspects that distinguish it from existing models. One of the primary innovations is its ability to discriminate between fibroblasts and tumor cells in co-cultures based on a combination of nuclear size, morphology, and association with collagen-1 staining. This allows for precise cell type differentiation without the need for multiple fibroblast markers, which are commonly required in other models. By avoiding reliance on extensive marker panels, our method simplifies the analysis process and increases its flexibility for a wide range of applications.

Additionally, a significant advantage of our approach is that both the immunostaining and image analysis are performed in 3D, ensuring that the entire volume of the sample is included in the analysis, rather than just a thin section as is common in conventional immunofluorescence staining of tissue slices. This provides a more comprehensive assessment of the cell interactions and spatial organization within the co-culture system, capturing the full complexity of the 3D tumor microenvironment. Traditional methods often lose critical spatial information when only 2D slices are analyzed.

Another important innovation of our 3D image analysis is the development of a custom Python script to quantify a wide array of features in 3D of the whole sample and at single-cell level. These enables the quantification of various cellular and morphological features in a highly automated and scalable manner, which has not been published in this form before. This allows for a deeper understanding of cellular behavior in complex environments, something that existing methods struggle to achieve in 3D co-cultures. The Python script is also compatible with the outputs of widely used deep-learning-based segmentation networks, such as Cellpose or Stardist. This compatibility means that users can easily integrate our analysis pipeline with the outputs of existing AI segmentation tools, offering flexibility in terms of input data. The script can be adapted to meet specific experimental needs, ensuring that segmentation masks generated by different AI tools can be used for downstream analysis. This flexibility makes our model not only novel but also highly adaptable to various experimental setups, enabling broader application in future research.

By combining advanced 3D immunostainings, deep-learning segmentation, and flexible software tools for 3D image analysis, the pipeline presented in this study represents a significant advancement over existing methods for co-culture analysis in 3D, particularly in terms of scalability, precision, and ease of use for studying complex tumor-stroma interactions.

However, one limitation of the current report is the absence of a cross-validation study using patient-derived cells. Although our results demonstrate the effectiveness of the proposed 3D co-culture model in drug testing, it remains to be explored how these findings translate to primary patient material. The use of patient-derived cells would provide a more clinically relevant evaluation of drug efficacy, aligning with the goal of developing personalized treatment strategies.

Future studies will focus on integrating patient-derived cells into our drug testing pipeline. This will allow us to match experimental results with clinical treatment outcomes, thus enabling the development of a more personalized approach to cancer therapy. By incorporating primary patient material, we aim to validate the robustness of our model in a clinical setting and further refine its utility for personalized medicine.

## Electronic Supplementary Material

Below is the link to the electronic supplementary material.

Supplementary Material 1

## Data Availability

Because of the large size of the imaging datasets shown in this paper, the datasets are not available in a public repository. They are available from the authors upon request. All code used in this protocol is provided on GitHub. (https://github.com/bruchr/Spheroid_Analysis).
