# Supplementary material for: A spheroid whole mount drug testing pipeline with machine-learning based image analysis identifies cell-type specific differences in drug efficacy on a single-cell level
Source: BMC Cancer. 2024 Dec 18;24:1542. doi: 10.1186/s12885-024-13329-9 (PMC11658419; doi:10.1186/s12885-024-13329-9)
Supplement: Supplementary file 1 — Supplementary Material 1 [file 12885_2024_13329_MOESM1_ESM.docx]

**Supplementary Material to Manuscript:**

**A spheroid whole mount drug testing pipeline with machine-learning based image analysis identifies cell-type specific differences in drug efficacy on a single-cell level**

**1. Supplementary Tables**

**Table S1: Quantitative results of KP4 and fibroblasts in mono- and co-culture treated with paclitaxel for 96h and 144 hours.** The readouts comprise the total number of nuclei, the volumes of spheroids and nuclei (both in µm^3^), the relative number of Ki-67^+^ cells in %, the relative changes in signal intensity of apoptotic cells indicated as signal sum cCaspase-3 / nuclei, the relative number of necrotic cells in %, the density of spheroids indicated as No. nuclei / µm^3^, and the relative spatial distribution of proliferative, apoptotic and necrotic cells of three biological replicates, each with six technical replicates. All values are given as mean ± SD. The significance levels, indicated by asterisks in the last column, show whether there were differences between the comparison of mono- vs co-cultures. Significance levels are denoted as follows: * p < 0.05, ** p < 0.01, *** p < 0.001, and **** p < 0.0001.

| Paclitaxel | | | | | | | | | |
| --- | --- | --- | --- | --- | --- | --- | --- | --- | --- |
| Readout | **Concentration / µM** | **KP4** | | | **KP4 +**  **CCD-1137Sk** | | | **CCD-1137Sk** | **p-Values**  **Mono- vs Co-culture** |
| Nuclei  96 hours | **0** | 9426 ± 1409 | | | 13580 ± 1492 | | | 1772 ± 115 | **** |
|  | **0.2** | 993 ± 151 | | | 2413 ± 72 | | | 1325 ± 105 | **** |
|  | **1** | 870 ± 124 | | | 1315 ± 98 | | | 939 ± 92 | **** |
|  | **5** | 1618 ± 46 | | | 1843 ± 208 | | | 1129 ± 119 | * |
| Nuclei  144 hours | **0** | 14727 ± 2797 | | | 20768 ± 1544 | | | 2026 ± 137 | *** |
|  | **0.2** | 415 ± 39 | | | 1035 ± 120 | | | 1198 ± 101 | *** |
|  | **1** | 375 ± 127 | | | 878 ± 47 | | | 647 ± 65 | *** |
|  | **5** | 663 ± 115 | | | 1247 ± 131 | | | 802 ± 103 | *** |
| Volume  Spheroids (µm^3^)  96 hours | **0** | 4.89E+07 ± 5.59E+05 | | | 6.63E+07 ± 2.03E+06 | | | 1.28E+06 ± 1.31E+04 | **** |
|  | **0.2** | 2.12E+06 ± 6.07E+04 | | | 2.87E+06 ± 7.59E+04 | | | 1.23E+06 ± 6.16E+04 | **** |
|  | **1** | 1.86E+06 ± 8.51E+04 | | | 2.45E+06 ± 1.13E+05 | | | 9.73E+05 ± 2.03E+04 | **** |
|  | **5** | 5.49E+06 ± 2.96E+05 | | | 5.81E+06 ± 1.32E+05 | | | 1.19E+06 ± 8.68E+04 | ns |
| Volume  Spheroids  144 hours | **0** | 7.61E+07 ± 2.07E+06 | | | 9.51E+07 ± 2.81E+06 | | | 1.35E+06 ± 6.51E+04 | ns |
|  | **0.2** | 8.75E+05 ± 1.62E+04 | | | 1.44E+06 ± 1.02E+05 | | | 1.18E+06 ± 7.44E+04 | **** |
|  | **1** | 7.06E+05 ± 7.17E+03 | | | 1.13E+06 ± 8.44E+04 | | | 8.58E+05 ± 3.74E+04 | **** |
|  | **5** | 1.25E+06 ± 5.27E+04 | | | 1.79E+06 ± 9.91E+04 | | | 1.08E+06 ± 5.87E+04 | **** |
| Volume  Nuclei (µm^3^)  96 hours | **0** | 1286 ± 50 | | | 1278 ± 63 | | | 511 ± 47 | ns |
|  | **0.2** | 1434 ± 55 | | | 909 ± 33 | | | 524 ± 56 | **** |
|  | **1** | 1472 ± 50 | | | 786 ± 23 | | | 476 ± 46 | **** |
|  | **5** | 1372 ± 76 | | | 943 ± 28 | | | 501 ± 72 | **** |
| Volume  Nuclei  144 hours | **0** | 1272 ± 76 | | | 1234 ± 41 | | | 466 ± 52 | ns |
|  | **0.2** | 1433 ± 48 | | | 910 ± 28 | | | 503 ± 63 | **** |
|  | **1** | 1417 ± 79 | | | 802 ± 39 | | | 502 ± 59 | **** |
|  | **5** | 1312 ± 21 | | | 933 ± 29 | | | 538 ± 55 | **** |
| Proliferation (%)  96 hours | **0** | 40.22 ± 5.2 | | | 50.83 ± 6.04 | | | 17.8 ± 2.58 | ** |
|  | **0.2** | 27.20 ± 3.89 | | | 17.67 ± 4.32 | | | 10.16 ± 2.13 | ** |
|  | **1** | 15.83 ± 2.22 | | | 6.25 ± 1.5 | | | 6.16 ± 1.94 | **** |
|  | **5** | 27.60 ± 2.96 | | | 19.75 ± 5.06 | | | 12.17 ± 2.14 | * |
| Proliferation  144 hours | **0** | 45.62 ± 5.51 | | | 57.50 ± 4.68 | | | 18.67 ± 3.82 | ** |
|  | **0.2** | 19.85 ± 3.33 | | | 10.80 ± 1.92 | | | 7.00 ± 2 | *** |
|  | **1** | 11.50 ± 2.64 | | | 5.16 ± 2.13 | | | 4.33 ± 1.50 | ** |
|  | **5** | 25.74 ± 3.87 | | | 25.24 ± 1.88 | | | 15.66 ± 2.88 | ns |
| Apoptosis  (signal sum cCaspase-3/nuclei)  96 hours | **0** | 83730 ± 7373 | | | 109027 ± 8249 | | | 14089 ± 2443 | ns |
|  | **0.2** | 237221 ± 30076 | | | 273569 ± 31881 | | | 36907 ± 5375 | ns |
|  | **1** | 253518 ± 45731 | | | 336700 ± 32705 | | | 48282 ± 4549 | *** |
|  | **5** | 217819 ± 22045 | | | 303117 ± 34926 | | | 37379 ± 4285 | *** |
| Apoptosis  144 hours | **0** | 119167 ± 10045 | | | 131864 ± 10845 | | | 18093 ± 2335 | ns |
|  | **0.2** | 312958 ± 48010 | | | 374058 ± 33401 | | | 51200 ± 6419 | ns |
|  | **1** | 347660 ± 19062 | | | 467208 ± 36162 | | | 69542 ± 2835 | **** |
|  | **5** | 238695 ± 19071 | | | 297114 ± 58390 | | | 46916 ± 6288 | ns |
| Necrosis (%)  96 hours | **0** | 8.43 ± 1.99 | | | 8.50 ± 3.39 | | | 4.00 ± 1.63 | ns |
|  | **0.2** | 18.86 ± 2.91 | | | 28.33 ± 6.18 | | | 8.66 ± 1.52 | ** |
|  | **1** | 31.14 ± 5.21 | | | 38.67 ± 3.26 | | | 17.50 ± 4.04 | * |
|  | **5** | 19.33 ± 2.73 | | | 23.14 ± 3.53 | | | 10.83 ± 2.78 | ns |
| Necrosis  144 hours | **0** | 14.83 ± 2.56 | | | 19.86 ± 2.85 | | | 8.17 ± 3.06 | ns |
|  | **0.2** | 27.83 ± 3.92 | | | 43.33 ± 5.39 | | | 16.3 ± 1.71 | **** |
|  | **1** | 40.00 ± 4.09 | | | 53.00 ± 4.94 | | | 22.8 ± 2.17 | *** |
|  | **5** | 23.75 ± 5.70 | | | 27.33 ± 4.22 | | | 16.5 ± 4.51 | ns |
| Density  (No. nuclei / µm^3^)  96 hours | **0** | 4.60E-04 ± 4.01E-05 | | | 4.73E-04 ± 3.44E-05 | | | 1.26E-03 ± 2.41E-05 | ns |
|  | **0.2** | 3.72E-04 ± 3.19E-05 | | | 8.71E-04 ± 1.74E-05 | | | 1.26E-03 ± 6.90E-05 | **** |
|  | **1** | 3.91E-04 ± 3.23E-05 | | | 7.91E-04 ± 4.55E-05 | | | 1.20E-03 ± 4.08E-05 | **** |
|  | **5** | 4.88E-04 ± 3.15E-05 | | | 5.53E-04 ± 2.51E-05 | | | 1.23E-03 ± 7.91E-05 | * |
| Density  144 hours | **0** | 4.59E-04 ± 3.42E-06 | | | 4.65E-04 ± 3.73E-05 | | | 1.24E-03 ± 2.96E-05 | ns |
|  | **0.2** | 3.48E-04 ± 3.25E-05 | | | 7.77E-04 ± 3.77E-05 | | | 1.28E-03 ± 2.40E-05 | **** |
|  | **1** | 3.56E-04 ± 2.45E-05 | | | 7.73E-04 ± 4.24E-05 | | | 1.19E-03 ± 5.56E-05 | **** |
|  | **5** | 5.05E-04 ± 4.41E-05 | | | 5.02E-04 ± 1.82E-05 | | | 1.29E-03 ± 3.24E-05 | ns |
| Shell Analysis (% / Ring) |  | **Inner Shell** | **Middle Shell** | **Outer Shell** | **Inner Shell** | **Middle Shell** | **Outer Shell** |  |  |
| Proliferation  96 hours | **0** | 11 | 27 | 62 | 13 | 21 | 66 |  |  |
|  | **0.2** | 20 | 37 | 43 | 4 | 18 | 78 |  |  |
|  | **1** | 17 | 39 | 44 | 2 | 16 | 82 |  |  |
|  | **5** | 8 | 41 | 51 | 6 | 23 | 71 |  |  |
| Proliferation  144 hours | **0** | 13 | 29 | 58 | 17 | 22 | 61 |  |  |
|  | **0.2** | 19 | 43 | 38 | 2 | 17 | 81 |  |  |
|  | **1** | 17 | 46 | 37 | 2 | 12 | 86 |  |  |
|  | **5** | 9 | 38 | 53 | 2 | 12 | 86 |  |  |
| Apoptosis  96 hours | **0** | 61 | 37 | 2 | 66 | 29 | 5 |  |  |
|  | **0.2** | 6 | 37 | 57 | 3 | 19 | 78 |  |  |
|  | **1** | 8 | 39 | 53 | 2 | 15 | 83 |  |  |
|  | **5** | 11 | 28 | 61 | 4 | 33 | 63 |  |  |
| Apoptosis  144 hours | **0** | 65 | 29 | 6 | 59 | 33 | 8 |  |  |
|  | **0.2** | 10 | 39 | 51 | 4 | 12 | 84 |  |  |
|  | **1** | 7 | 37 | 56 | 2 | 14 | 84 |  |  |
|  | **5** | 11 | 25 | 64 | 3 | 28 | 69 |  |  |
| Necrosis  96 hours | **0** | 53 | 42 | 5 | 57 | 32 | 11 |  |  |
|  | **0.2** | 11 | 22 | 67 | 1 | 11 | 88 |  |  |
|  | **1** | 5 | 36 | 59 | 2 | 12 | 86 |  |  |
|  | **5** | 17 | 39 | 44 | 6 | 27 | 67 |  |  |
| Necrosis  144 hours | **0** | 43 | 48 | 9 | 55 | 35 | 10 |  |  |
|  | **0.2** | 7 | 32 | 61 | 3 | 13 | 84 |  |  |
|  | **1** | 4 | 28 | 68 | 4 | 15 | 81 |  |  |
|  | **5** | 12 | 37 | 51 | 9 | 19 | 72 |  |  |

**Table S2: Quantitative results of KP4 and fibroblasts in mono- and co-culture treated with doxorubicin for 96h and 144 hours.** The readouts comprise the total number of nuclei, the volumes of spheroids and nuclei (both in µm^3^), the relative number of Ki-67+ cells in %, the relative changes in signal intensity of apoptotic cells indicated as signal sum cCaspase-3 / nuclei, the relative number of necrotic cells in %, the density of spheroids indicated as No. nuclei / µm^3^, and the relative spatial distribution of proliferative, apoptotic and necrotic cells of three biological replicates, each with six technical replicates. All values are given as mean ± SD. The significance levels, indicated by asterisks in the last column, show whether there were differences between the comparison of mono- vs co-cultures. Significance levels are denoted as follows: * p < 0.05, ** p < 0.01, *** p < 0.001, and **** p < 0.0001.

| Doxorubicin | | | | | | | | | |
| --- | --- | --- | --- | --- | --- | --- | --- | --- | --- |
| Readout | **Concentration / µM** | **KP4** | | | **KP4 +**  **CCD-1137Sk** | | | **CCD-1137Sk** | **p-Values**  **Mono- vs Co-culture** |
| Nuclei  96 hours | **0** | 8742 ± 497 | | | 12421 ± 1522 | | | 1860 ± 119 | **** |
|  | **0.2** | 1835 ± 230 | | | 2859 ± 267 | | | 943 ± 76 | *** |
|  | **1** | 1111 ± 110 | | | 1576 ± 115 | | | 583 ± 64 | ** |
|  | **5** | 422 ± 67 | | | 488 ± 78 | | | 304 ± 72 | ns |
| Nuclei  144 hours | **0** | 15348 ± 852 | | | 21629 ± 642 | | | 2130 ± 230 | **** |
|  | **0.2** | 717 ± 15 | | | 859 ± 94 | | | 717 ± 15 | *** |
|  | **1** | 611 ± 93 | | | 777 ± 145 | | | 611 ± 93 | ** |
|  | **5** | 142 ± 25 | | | 164 ± 13 | | | 142 ± 25 | ns |
| Volume  Spheroids (µm^3^)  96 hours | **0** | 4.84E+07 ± 1.54E+06 | | | 6.46E+07 ± 3.36E+06 | | | 1.27E+06 ± 1.16E+04 | **** |
|  | **0.2** | 8.52E+06 ± 2.99E+05 | | | 1.12E+07 ± 7.12E+05 | | | 8.45E+05 ± 4.35E+04 | **** |
|  | **1** | 2.54E+06 ± 7.80E+04 | | | 3.21E+06 ± 1.67E+05 | | | 4.38E+05 ± 3.68E+04 | **** |
|  | **5** | 5.20E+05 ± 1.62E+04 | | | 5.02E+05 ± 2.09E+04 | | | 2.34E+05 ± 2.63E+04 | ns |
| Volume  144 hours | **0** | 7.56E+07 ± 2.77E+06 | | | 9.62E+07 ± 2.72E+06 | | | 1.34E+06 ± 3.85E+04 | **** |
|  | **0.2** | 1.63E+06 ± 9.12E+04 | | | 1.82E+06 ± 6.82E+04 | | | 7.22E+05 ± 4.31E+04 | * |
|  | **1** | 1.25E+06 ± 5.74E+04 | | | 1.41E+06 ± 3.30E+04 | | | 6.29E+05 ± 2.40E+04 | ** |
|  | **5** | 1.66E+05 ± 4.66E+03 | | | 1.67E+05 ± 6.79E+03 | | | 1.59E+05 ± 1.18E+04 | ns |
| Volume  Nuclei (µm^3^)  96 hours | **0** | 1239 ± 79 | | | 1230 ± 40 | | | 506 ± 65 | ns |
|  | **0.2** | 1231 ± 54 | | | 881 ± 35 | | | 524 ± 62 | **** |
|  | **1** | 1257 ± 37 | | | 869 ± 51 | | | 480 ± 520 | **** |
|  | **5** | 1217 ± 40 | | | 852 ± 74 | | | 555 ± 61 | **** |
| Volume  144 hours | **0** | 1240 ± 39 | | | 1257 ± 50 | | | 537 ± 69 | ns |
|  | **0.2** | 1248 ± 52 | | | 896 ± 42 | | | 542 ± 93 | **** |
|  | **1** | 1276 ± 49 | | | 844 ± 66 | | | 482 ± 42 | **** |
|  | **5** | 1259 ± 82 | | | 841 ± 37 | | | 524 ± 41 | **** |
| Proliferation (%)  96 hours | **0** | 43.24 ± 3.44 | | | 53.50 ± 4.50 | | | 16.60 ± 4.39 | ** |
|  | **0.2** | 26.79 ± 1.24 | | | 20.43 ± 3.55 | | | 10.80 ± 3.34 | *** |
|  | **1** | 20.24 ± 1.48 | | | 8.86 ± 2.19 | | | 4.67 ± 1.96 | **** |
|  | **5** | 8.27 ± 1.59 | | | 2.99 ± 0.99 | | | 2.50 ± 1.04 | ** |
| Proliferation  144 hours | **0** | 44.91 ± 6.78 | | | 52.40 ± 2.70 | | | 22.00 ± 3.34 | * |
|  | **0.2** | 21.17 ± 2.78 | | | 13.50 ± 3.0 | | | 7.33 ± 2.80 | ** |
|  | **1** | 11.17 ± 2.04 | | | 3.33 ± 0.57 | | | 3.10 ± 1.41 | *** |
|  | **5** | 4.03 ± 0.95 | | | 0.95 ± 0.53 | | | 1.60 ± 0.54 | ** |
| Apoptosis  (signal sum cCaspase-3/nuclei)  96 hours | **0** | 87811 ± 6707 | | | 104220 ± 13238 | | | 13489 ± 1061 | ns |
|  | **0.2** | 205865 ± 57884 | | | 245153 ± 37221 | | | 58999 ± 3536 | ns |
|  | **1** | 293616 ± 42269 | | | 384536 ± 53776 | | | 95302 ± 7847 | ** |
|  | **5** | 399195 ± 34137 | | | 589932 ± 47684 | | | 117947 ± 17778 | **** |
| Apoptosis  144 hours | **0** | 123280 ± 13957 | | | 135127 ± 10316 | | | 19186 ± 2298 | ns |
|  | **0.2** | 287204 ± 44141 | | | 437629 ± 49216 | | | 80190 ± 6221 | * |
|  | **1** | 380345 ± 56000 | | | 559972 ± 60457 | | | 147756 ± 8425 | ** |
|  | **5** | 707586 ± 99692 | | | 752481 ± 146759 | | | 153867 ± 37601 | ns |
| Necrosis (%)  96 hours | **0** | 8.33 ± 1.86 | | | 8.66 ± 1.63 | | | 5.50 ± 1.05 | ns |
|  | **0.2** | 16.50 ± 3.27 | | | 26.17 ± 3.18 | | | 17.6 ± 5.13 | * |
|  | **1** | 24.86 ± 4.10 | | | 35.00 ± 4.10 | | | 28.7 ± 4.41 | * |
|  | **5** | 52.60 ± 7.98 | | | 61.80 ± 7.79 | | | 45.2 ± 8.70 | ns |
| Necrosis  144 hours | **0** | 12.29 ± 3.68 | | | 20.67 ± 1.96 | | | 8.00 ± 2.12 | ns |
|  | **0.2** | 22.80 ± 3.76 | | | 35.86 ± 3.93 | | | 31.1 ± 2.34 | **** |
|  | **1** | 35.33 ± 5.53 | | | 46.67 ± 5.01 | | | 43.5 ± 8.83 | ** |
|  | **5** | 54.33 ± 11.55 | | | 70.17 ± 2.64 | | | 53.8 ± 9.26 | ** |
| Density  (No. nuclei / µm^3^)  96 hours | **0** | 4.73E-04 ± 3.33E-05 | | | 4.53E-04 ± 2.77E-05 | | | 1.29E-03 ± 3.40E-05 | ns |
|  | **0.2** | 2.57E-04 ± 1.60E-05 | | | 2.59E-04 ± 1.70E-05 | | | 1.24E-03 ± 1.93E-05 | ns |
|  | **1** | 5.05E-04 ± 2.01E-05 | | | 5.14E-04 ± 5.61E-05 | | | 1.27E-03 ± 2.38E-05 | ns |
|  | **5** | 6.44E-04 ± 3.08E-05 | | | 4.62E-04 ± 1.74E-05 | | | 5.40E-04 ± 3.37E-05 | **** |
| Density  144 hours | **0** | 4.51E-04 ± 3.35E-05 | | | 4.70E-04 ± 3.14E-05 | | | 1.29E-03 ± 2.25E-05 | ns |
|  | **0.2** | 3.85E-04 ± 3.43E-05 | | | 3.06E-04 ± 4.47E-05 | | | 1.23E-03 ± 5.82E-05 | ** |
|  | **1** | 4.86E-04 ± 3.90E-05 | | | 4.58E-04 ± 1.62E-05 | | | 1.23E-03 ± 4.26E-05 | ns |
|  | **5** | 6.71E-04 ± 3.50E-05 | | | 4.94E-04 ± 1.51E-05 | | | 5.56E-04 ± 6.72E-05 | *** |
| Shell Analysis (% / Ring) |  | **Inner Shell** | **Middle Shell** | **Outer Shell** | **Inner Shell** | **Middle Shell** | **Outer Shell** |  |  |
| Proliferation  96 hours | **0** | 13 | 28 | 59 | 15 | 33 | 52 |  |  |
|  | **0.2** | 16 | 44 | 40 | 4 | 24 | 72 |  |  |
|  | **1** | 9 | 38 | 53 | 1 | 13 | 86 |  |  |
|  | **5** | 2 | 46 | 52 | 0 | 9 | 91 |  |  |
| Proliferation  144 hours | **0** | 12 | 26 | 62 | 17 | 29 | 54 |  |  |
|  | **0.2** | 14 | 48 | 38 | 6 | 21 | 73 |  |  |
|  | **1** | 11 | 52 | 47 | 2 | 15 | 83 |  |  |
|  | **5** | 2 | 41 | 57 | 1 | 11 | 88 |  |  |
| Apoptosis  96 hours | **0** | 57 | 37 | 6 | 61 | 35 | 4 |  |  |
|  | **0.2** | 12 | 36 | 52 | 9 | 29 | 62 |  |  |
|  | **1** | 15 | 43 | 42 | 19 | 42 | 39 |  |  |
|  | **5** | 27 | 34 | 39 | 34 | 30 | 36 |  |  |
| Apoptosis  144 hours | **0** | 61 | 35 | 4 | 55 | 40 | 5 |  |  |
|  | **0.2** | 14 | 42 | 44 | 12 | 30 | 58 |  |  |
|  | **1** | 11 | 38 | 51 | 17 | 36 | 47 |  |  |
|  | **5** | 11 | 38 | 51 | 29 | 28 | 43 |  |  |
| Necrosis  96 hours | **0** | 57 | 39 | 4 | 65 | 33 | 2 |  |  |
|  | **0.2** | 13 | 28 | 59 | 14 | 34 | 52 |  |  |
|  | **1** | 12 | 41 | 47 | 22 | 32 | 46 |  |  |
|  | **5** | 31 | 37 | 33 | 28 | 38 | 34 |  |  |
| Necrosis  144 hours | **0** | 52 | 46 | 2 | 59 | 36 | 5 |  |  |
|  | **0.2** | 10 | 33 | 57 | 16 | 37 | 47 |  |  |
|  | **1** | 15 | 39 | 46 | 30 | 29 | 41 |  |  |
|  | **5** | 36 | 31 | 33 | 34 | 39 | 27 |  |  |

**Table S3: Corrected values for KP4 + CCD1137Sk co-cultures treated with paclitaxel and doxorubicin for 96h and 144 hours indicate the individual numbers of KP4 and CCD1137Sk within co-cultures** Absolute numbers of nuclei from KP4 mono- and co-cultures treated with paclitaxel and doxorubicin at 96h and 144h of three biological replicates, each with six technical replicates. Corrected values from co-cultures are showing the quantity of KP4 tumor cells and CCD1137Sk fibroblasts individually within the co-cultures and were calculated by subtracting predicted fibroblast nuclei from total nuclei numbers of the corresponding co-culture group (see Table S1-S2). Values are given as mean ± SD.

| Paclitaxel Corrected Nuclei | | | |
| --- | --- | --- | --- |
| 96h | **KP4 Mono-culture** | **KP4 + CCD-1137Sk Co-culture** | |
| Concentration / µM | **KP4** | **KP4 Nuclei** | **CCD-1137Sk Nuclei** |
| 0 | 9426 ± 1409 | 12249 ± 668 | 1330 ± 445 |
| 0.2 | 993 ± 151 | 1341 ± 152 | 1072 ± 145 |
| 1 | 870 ± 124 | 401 ± 46 | 914 ± 83 |
| 5 | 1618 ± 46 | 677 ± 65 | 1166 ± 216 |
|  |  |  |  |
| 144h |  |  |  |
| Concentration / µM | **KP4** | **KP4 Nuclei** | **CCD-1137Sk Nuclei** |
| 0 | 14727 ± 2797 | 19364 ± 1261 | 1403 ± 369 |
| 0.2 | 415 ± 39 | 384 ± 62 | 651 ± 82 |
| 1 | 375 ± 127 | 299 ± 51 | 579 ± 40 |
| 5 | 663 ± 115 | 484 ± 121 | 763 ± 77 |
|  |  |  |  |
| Doxorubicin Corrected Nuclei | | | |
| 96h | **KP4 Mono-culture** | **KP4 + CCD-1137Sk Co-culture** | |
| Concentration / µM | **KP4** | **KP4 Nuclei** | **CCD-1137Sk Nuclei** |
| 0 | 8742 ± 497 | 11183 ± 1413 | 1238 ± 168 |
| 0.2 | 1835 ± 230 | 1784 ± 248 | 1076 ± 60 |
| 1 | 1111 ± 110 | 700 ± 157 | 876 ± 117 |
| 5 | 422 ± 67 | 212 ± 96 | 276 ± 55 |
|  |  |  |  |
| 144h |  |  |  |
| Concentration / µM | **KP4** | **KP4 Nuclei** | **CCD-1137Sk Nuclei** |
| 0 | 15348 ± 852 | 20400 ± 607 | 1229 ± 374 |
| 0.2 | 717 ± 15 | 399 ± 63 | 460 ± 113 |
| 1 | 611 ± 93 | 454 ± 185 | 323 ± 71 |
| 5 | 142 ± 25 | 68 ± 3 | 96 ± 15 |
|  |  |  |  |

**Table S4**: The most important metrics generated from the Python script:

**Spheroid Features:**

| Spheroid volume | Volume of the whole spheroid calculation based on nuclei segmentation. |
| --- | --- |
| Spheroid volume void | Volume inside the spheroid, but without nuclei (Spheroid volume – Nuclei volume) |
| Spheroid void ratio | Ratio of the void volume to the complete spheroid volume. This feature gives an overview, how much volume is occupied of the nuclei. |
| Spheroid equivalent diameter | Diameter of a circle with the same area as the maximum area of an x-y plane. This feature is useful to compare diameter measurements performed in bright field. |
| Nuclei density | Density is approximated by dividing the number of nuclei inside the cell culture by the spheroid volume. This feature is similar to the spheroid void ratio, but is independent of the nuclei volumes. |
| Number of nuclei | Number of nuclei in the image |
| Number of positive/negative nuclei | If a classification model is supplied, the number of positive/negative classified nuclei is calculated. |
| Mean nuclei volume | The mean volume of nuclei. |
| Mean nuclei distance to hull | The mean distance of nuclei centroids to the cell culture hull. |
| Mean nuclei distance to hull of positive/negative nuclei | If a classification model is supplied, the distance of positive/negative classified nuclei to the cell culture hull is calculated. |
| Mean nuclei distance to spheroid center | The mean distance of nuclei centroids to the center of the cell culture. |
| Mean nuclei distance to spheroid center of positive/negative nuclei | If a classification model is supplied, the distance of positive/negative classified nuclei to the cell culture center is calculated. |
| Signal mean | The mean signal of the complete image for each channel. |
| Signal mean foreground | The mean signal of only the foreground region for each channel. The foreground region is determined by user-defined thresholds. This is useful to suppress background areas. |
|  |  |
| Shell analysis: Volume | Volume of each shell. |
| Shell analysis: Nuclei density | Number of nuclei divided by the volume of the shell. |
| Shell analysis: Number of nuclei | Number of nuclei located in each shell. |
| Shell analysis: Number of positive/negative nuclei | If a classification model is supplied, the number of positive/negative classified nuclei inside each shell is calculated. |
| Shell analysis: Mean nuclei volume | The mean volume of nuclei inside each shell. This feature enables the analysis if nuclei located deep inside the cell culture feature a lower volume. |
| Shell analysis: Signal mean | Mean signal inside each shell calculated for each channel |
|  |  |
| Shell analysis: Signal mean foreground | Mean signal of foreground regions inside each shell and for all channels. The foreground region is determined by user-defined thresholds. |
| Additional Object analysis: | If additional segmentation masks are provided the following features are extracted. |
| Number of objects | Number of objects in the provided segmentation mask. |
| Mean object distance to spheroid hull | Mean distance between object centroids and the spheroid hull. |
| Mean object distance to spheroid center | Mean distance between object centroids and the center of the spheroid. |
| Shell analysis: Number of objects | Number of objects located in each shell. |

**Nuclei Features:**

| Nuclei volume | Volume of the nuclei. |
| --- | --- |
| Distance to hull | Distance of the nuclei center to the closest point on the hull. |
| Distance to spheroid center | Distance of the nuclei center to the center of the cell culture. |
| Shell location | ID of the shell the nuclei is located in. |
| Centroid position | Centroid coordinated of the nuclei (z,y,x). |
| Extend | Ratio of nuclei volume to bounding box volume. |
| Segmentation label number | Label ID of the nuclei in the provided segmentation mask. |
| Major / Minor axis length | Axis length of an ellipsoid fitted to the nuclei label. |
| Moments | Spacial moments of the nuclei label. |
| Solidity | Ratio of nuclei volume to the volume of its convex hull. |
| Cell type (pos or neg) | If a classification model is supplied, the class of the nuclei is given. |
| Mean intensity | Mean intensity inside the nuclei is calculated for each channel. |

**2. Supplementary Figures**

**
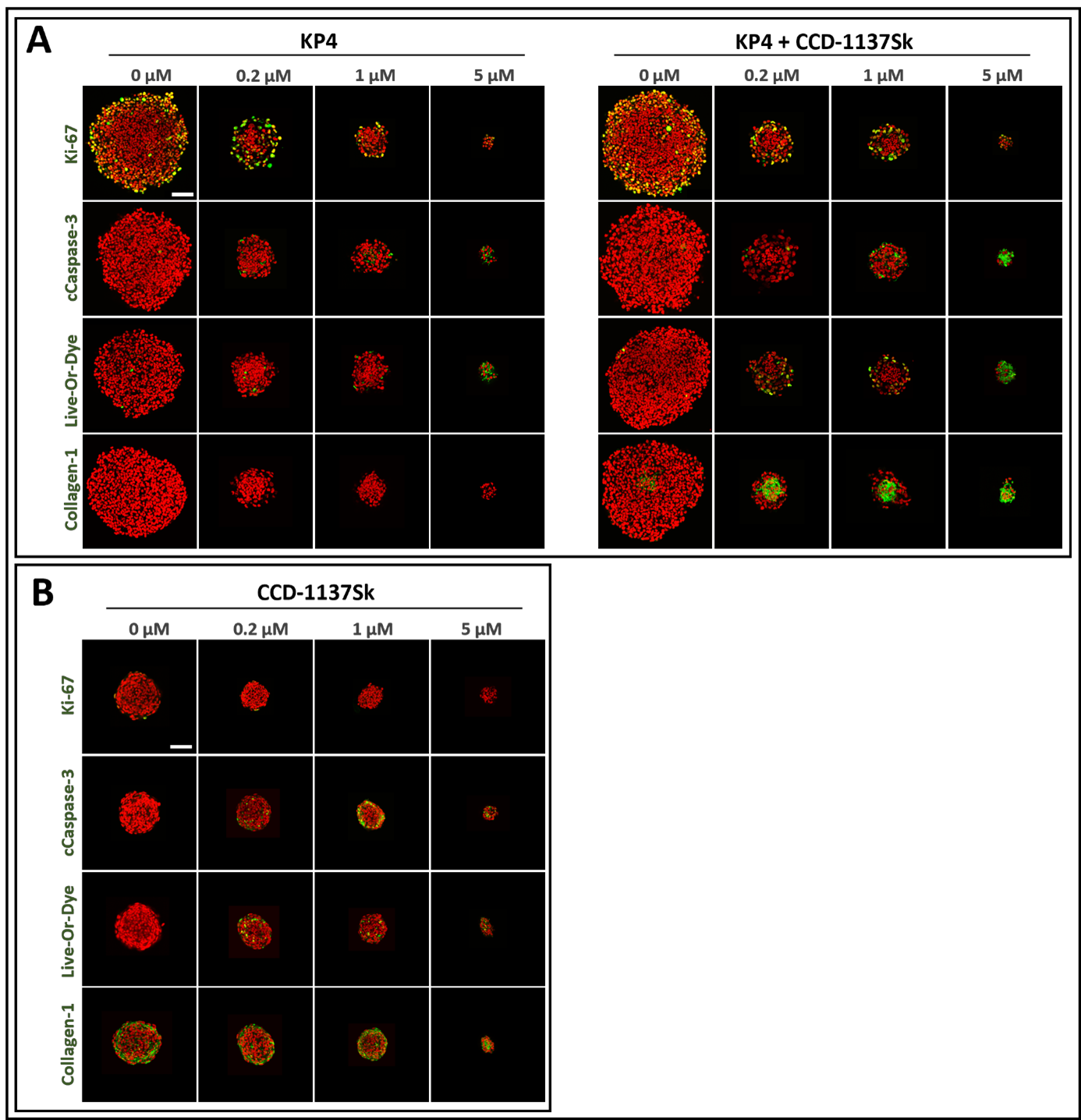
**

**Figure S1:** **Doxorubicin treatment exhibits differential effects on KP4 cancer cells and CCD-1137Sk fibroblasts.** KP4 tumor cells and CCD-1137Sk fibroblasts were seeded into 96-well ULA plates in mono- and co-culture and treated with doxorubicin after three days in culture for 96 h at four different concentrations (0, 0.2, 1, and 5 µM). Then, spheroid whole mounts were fixed, cleared, and stained with fluorescence markers to detect proliferation (Ki-67), apoptosis (Cleaved Caspase-3), necrosis (Live-or-Dye), fibroblasts (Collagen-1), and nuclei (DRAQ5). Whole mount 3D-confocal microscopy and CNN-based 3D-image segmentation was performed. All scalebars, 100 µm. **(A-B)** Representative micrographs showing single optical sections through spheroids in KP4 mono- **(A, left panels)** or co-culture **(A, right panels)** or CCD-1137Sk mono-culture **(B)** at the largest spheroid circumference with indicated fluorescence markers. Nuclei (red) and marker signals (green) are shown as overlays.

**
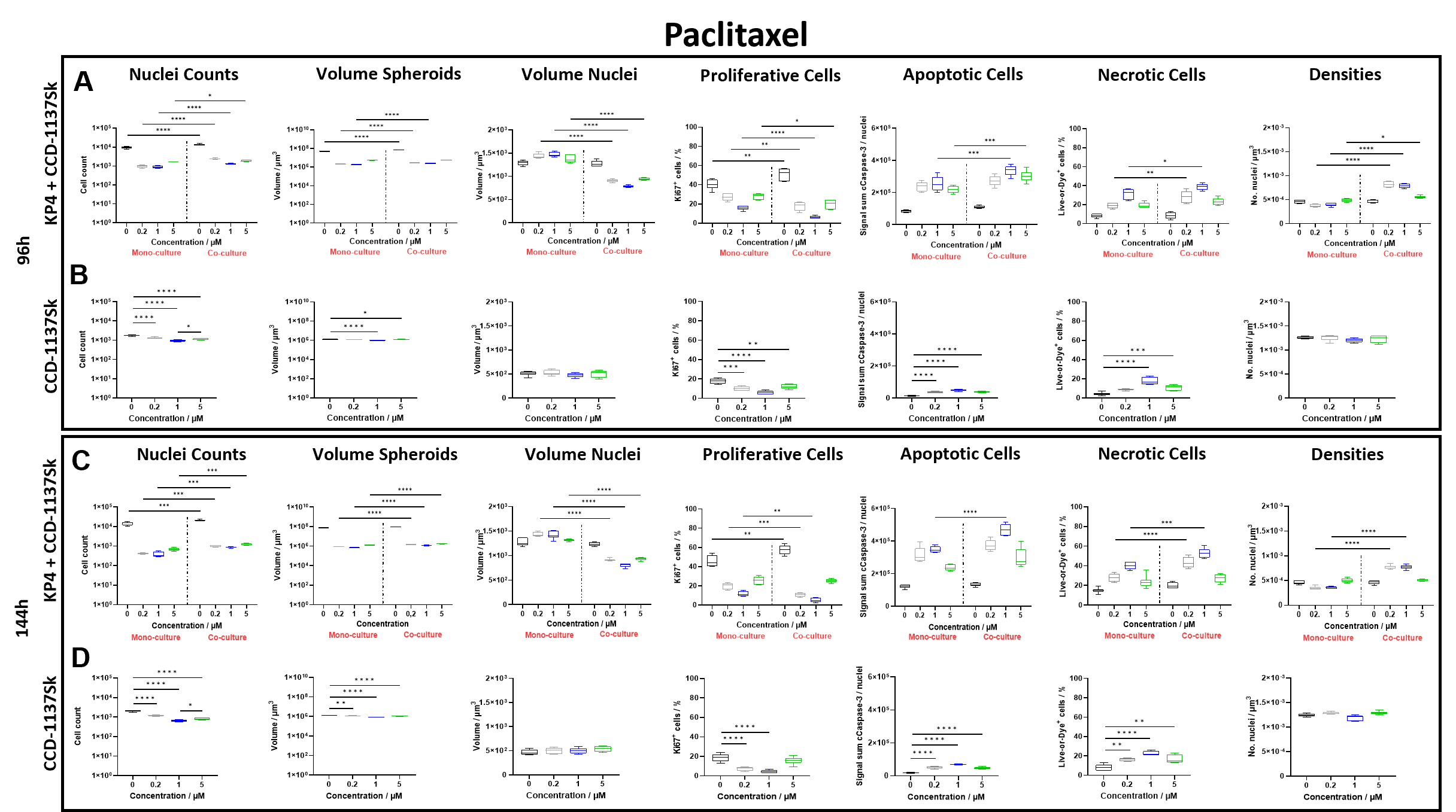
**

**Figure S2: Quantitative results of KP4 and fibroblasts in mono- and co-culture treated with paclitaxel for 96 and 144 hours.** The Box plots depicting the total number of nuclei, the volumes of spheroids and nuclei (both in µm^3^), the relative number of Ki-67+ cells in %, the relative changes in signal intensity of apoptotic cells indicated as signal sum cCaspase-3 / nuclei, the relative number of necrotic cells in %, and the density of spheroids indicated as No. nuclei / µm^3^ of three biological replicates, each with six technical replicates (mean ± SD, 25- and 75-percentiles are plotted). The graphs for the number of nuclei and spheroid volumes have logarithmic scaling on the y-axes (log10) because of the wide range of values. The significance levels, indicated by asterisks in the last column, show whether there were differences between the comparison of mono- vs co-cultures. Significance levels are denoted as follows: * p < 0.05, ** p < 0.01, *** p < 0.001, and **** p < 0.0001.

**
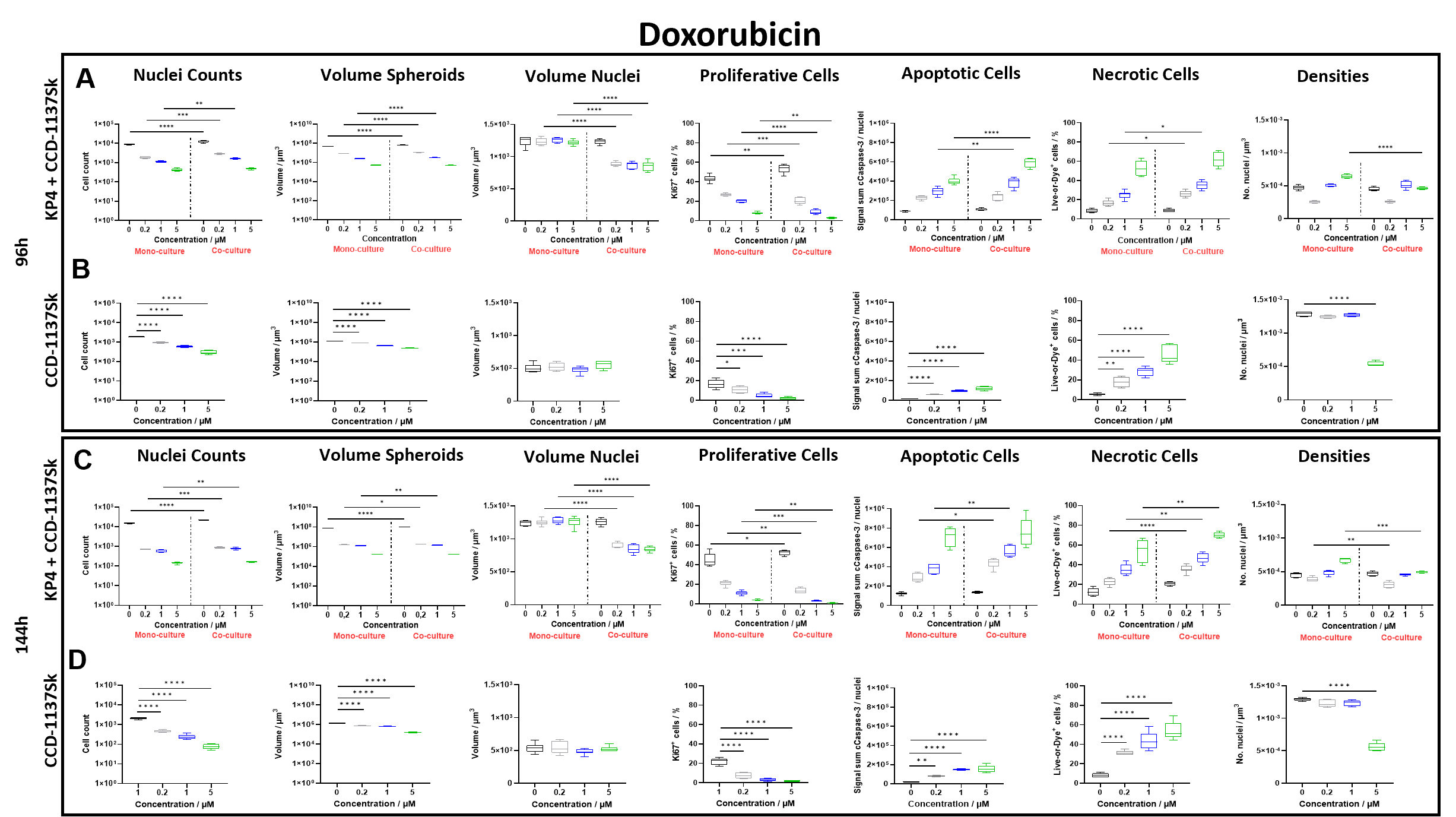
**

**Figure S3: Quantitative results of KP4 and fibroblasts in mono- and co-culture treated with doxorubicin for 96 and 144 hours.** The Box plots depicting the total number of nuclei, the volumes of spheroids and nuclei (both in µm^3^), the relative number of Ki-67+ cells in %, the relative changes in signal intensity of apoptotic cells indicated as signal sum cCaspase-3 / nuclei, the relative number of necrotic cells in %, and the density of spheroids indicated as No. nuclei / µm^3^ of three biological replicates, each with six technical replicates (mean ± SD, 25- and 75-percentiles are plotted). The graphs for the number of nuclei and spheroid volumes have logarithmic scaling on the y-axes (log10) because of the wide range of values. The significance levels, indicated by asterisks in the last column, show whether there were differences between the comparison of mono- vs co-cultures. Significance levels are denoted as follows: * p < 0.05, ** p < 0.01, *** p < 0.001, and **** p < 0.0001.

**
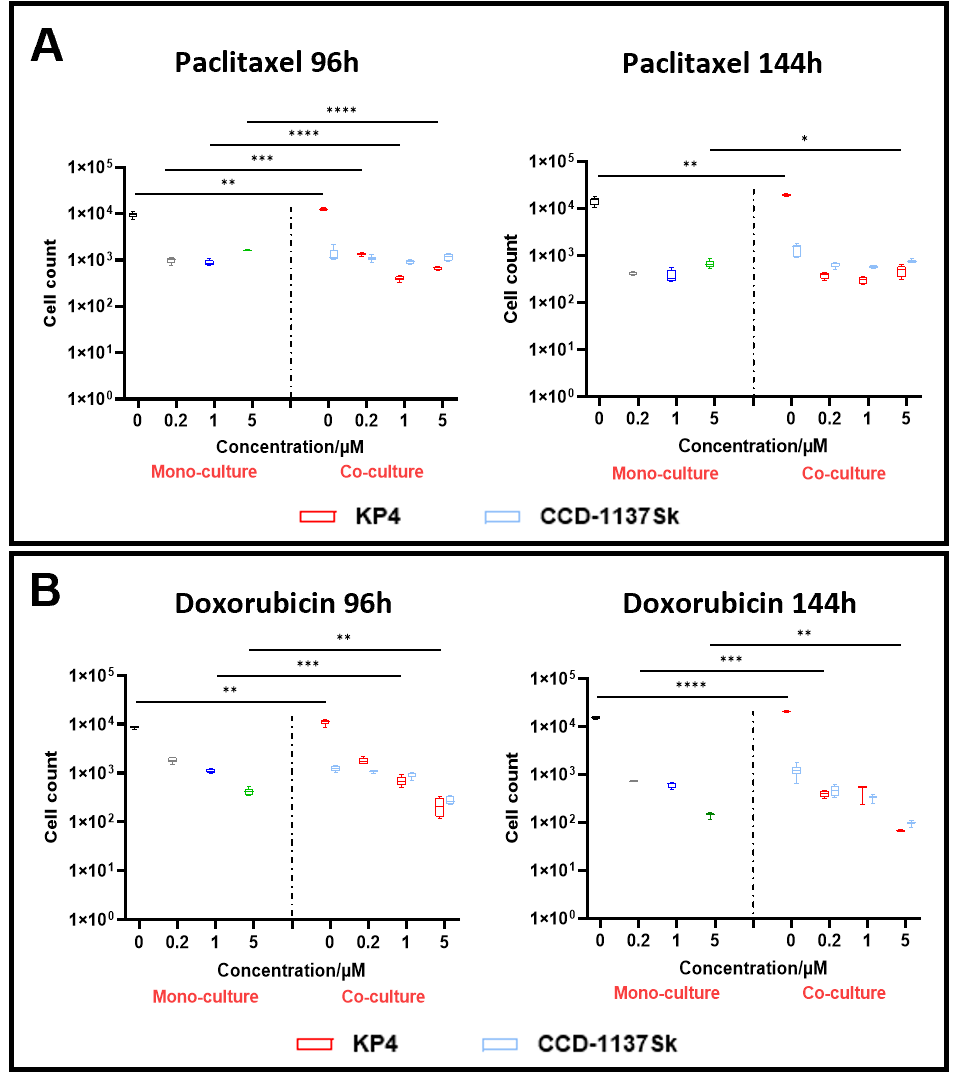
**

**Figure S4:** **Corrected values for KP4 + CCD1137Sk co-cultures treated with paclitaxel and doxorubicin for 96 and 144 hours indicate the individual numbers of KP4 and CCD1137Sk within co-cultures.** Box plots show absolute numbers of nuclei from KP4 mono- and co-cultures treated with paclitaxel and doxorubicin at 96h and 144h of three biological replicates, each with six technical replicates (mean ± SD, 25- and 75-percentiles are plotted). Corrected values from co-cultures are showing the quantity of KP4 tumor cells (red) and CCD1137Sk fibroblasts (blue) individually within the co-cultures and were calculated by subtracting predicted fibroblast nuclei from total nuclei numbers of the corresponding co-culture group (see Table S1-S2).

**
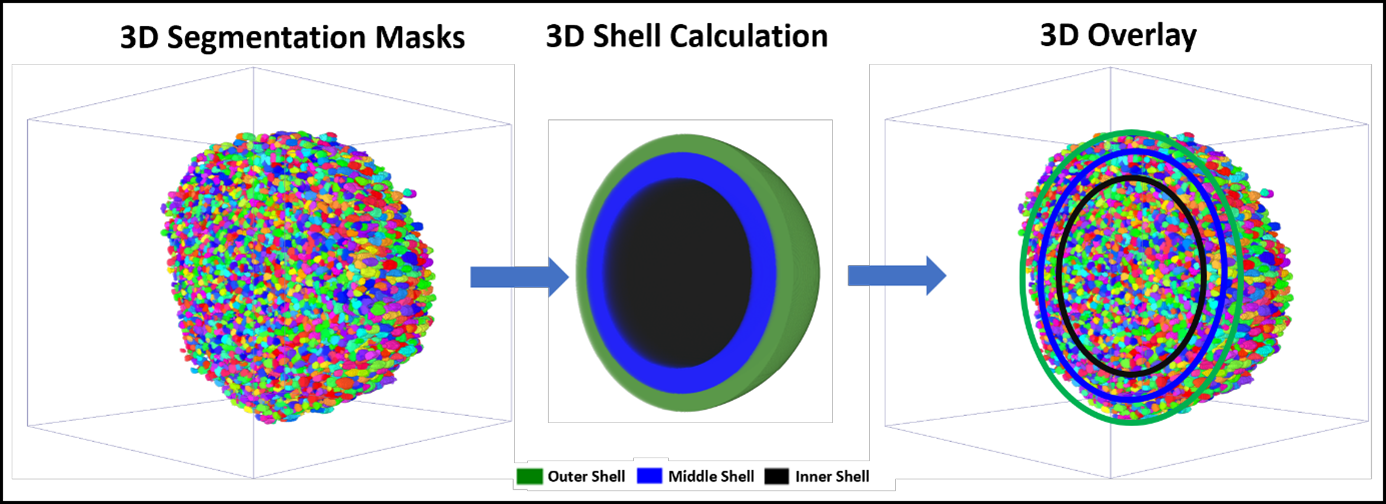
**

**Figure S5:** **Schematic workflow of the 3D shell analysis**. Segmentation masks were used as input for the 3D shell analysis. The masks were initially divided into three shells, each with the same volume: an inner, middle, and outer shell. This division was achieved by applying binary erosion operations to the entire structure, gradually eroding the structure from the outside inwards. After each erosion, the remaining volume was calculated and repeated until the remaining volume of the spheroid was reduced to two thirds and then to one third of the original volume.

**
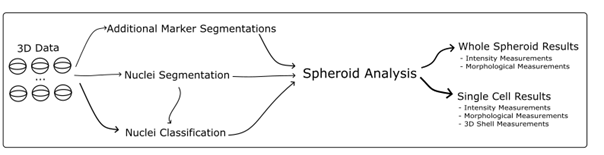
**

**Figure S6: Workflow diagram of 3D spheroid analysis.** The scheme details the sequential processes of nuclei segmentation and classification, followed by comprehensive data acquisition for whole spheroid and single cell results, including intensity, morphological, and 3D shell measurements.
